# TagP, a PAAR-domain containing protein, plays roles in the fitness and virulence of *Acinetobacter baumannii*

**DOI:** 10.3389/fcimb.2024.1379106

**Published:** 2024-07-18

**Authors:** Yanbing Li, Yiming Cui, Kai Song, Leiming Shen, Liting Xiao, Junyan Jin, Yanting Zhao, Yanfeng Yan, Shengyuan Zhao, Wenwu Yao, Shihua Wang, Zongmin Du, Ruifu Yang, Bin Yi, Yajun Song

**Affiliations:** ^1^ State Key Laboratory of Pathogen and Biosecurity, Beijing Institute of Microbiology and Epidemiology, Beijing, China; ^2^ Department of Clinical Laboratory, Xiangya Hospital, Central South University, Changsha, Hunan, China; ^3^ School of Life Sciences, Fujian Agriculture and Forestry University, Fuzhou, China

**Keywords:** *A. baumannii*, type VI secretion system, PAAR protein, histone-like nucleoid structuring protein, environmental fitness, virulence

## Abstract

**Background:**

Type VI secretion system (T6SS) is widely present in Gram-negative bacteria and directly mediates antagonistic prokaryote interactions. PAAR (proline-alanine-alanine-arginine repeats) proteins have been proven essential for T6SS-mediated secretion and target cell killing. Although PAAR proteins are commonly found in *A. baumannii*, their biological functions are not fully disclosed yet. In this study, we investigated the functions of a PAAR protein termed TagP (T6SS-associated-gene PAAR), encoded by the gene ACX60_RS09070 outside the core T6SS locus of *A. baumannii* strain ATCC 17978.

**Methods:**

In this study, *tagP* null and complement *A. baumannii* ATCC 17978 strains were constructed. The influence of TagP on T6SS function was investigated through Hcp detection and bacterial competition assay; the influence on environmental fitness was studied through in vitro growth, biofilm formation assay, surface motility assay, survivability in various simulated environmental conditions; the influence on pathogenicity was explored through cell adhesion and invasion assays, intramacrophage survival assay, serum survival assay, and *G. melonella* Killing assays. Quantitative transcriptomic and proteomic analyses were utilized to observe the global impact of TagP on bacterial status.

**Results:**

Compared with the wildtype strain, the *tagP* null mutant was impaired in several tested phenotypes such as surface motility, biofilm formation, tolerance to adverse environments, adherence to eukaryotic cells, endurance to serum complement killing, and virulence to *Galleria melonella*. Notably, although RNA-Seq and proteomics analysis revealed that many genes were significantly down-regulated in the *tagP* null mutant compared to the wildtype strain, there is no significant difference in their antagonistic abilities. We also found that Histone-like nucleoid structuring protein (H-NS) was significantly upregulated in the *tagP* null mutant at both mRNA and protein levels.

**Conclusions:**

This study enriches our understanding of the biofunction of PAAR proteins in *A. baumannii*. The results indicates that TagP involved in a unique modulation of fitness and virulence control in *A. baumannii*, it is more than a classic PAAR protein involved in T6SS, while how TagP play roles in the fitness and virulence of *A. baumannii* needs further investigation to clarify.

## 1 Introduction

*Acinetobacter baumannii* is a very important pathogen responsible for nosocomial infections and notorious for its antibiotic resistance profiles (Ayobami et al., 2019; Hong et al., 2020). *A. baumannii* poses a significant threat to public healthcare (Gu et al., 2022; Gao and Wang, 2023). The ability of *A. baumannii* to occupy favorable ecological niches and adhere to eukaryotic cells, along with its desiccation tolerance and biofilm formation, contribute to its survival in harsh environments.

Type VI secretion system (T6SS) has been identified in around 25% of Gram-negative bacteria and serves as a close-range competition weapon for many bacteria (Boyer et al., 2009). It also plays a crucial role in bacterial virulence, general stress responses, and resistance to inherent host immunity (Zhao et al., 2018; Wood et al., 2019a; Corral et al., 2021). In *A. baumannii*, the core T6SS locus consists of 13 conserved core genes (*tss* genes), along with T6SS-associated genes (*tag* genes) and T6SS effector genes (*tse* genes) (Weber et al., 2016). Haemolysin co-regulated protein (Hcp) is also an important component of the T6SS and serves as an indicator of active T6SS when detected in culture supernatant (Ma et al., 2018).

Located within or outside the core T6SS gene cluster, PAAR (proline-alanine-alanine-arginine repeats) proteins can enhance the puncturing ability of T6SS by interacting with the VgrG (valine-glycine repeat G) proteins at the tip of T6SS (Shneider et al., 2013) and have diverse functions through various C-terminal extension domains (Cianfanelli et al., 2016; Liu et al., 2021). A bioinformatic analysis of 97 *A. baumannii* genomes revealed that 91 of the strains contained 1 to 4 genes encoding PAAR proteins, while 6 of the strains lacked such genes (5 of which lacked complete T6SS gene clusters) (Lewis et al., 2019). This study identified 13 types (A to M) PAAR proteins based on their amino acid sequences. The shortest but most abundant PAAR-M protein is always present in the core T6SS locus and is almost exclusively found in strains that also encode Hcp protein. Other types of PAAR proteins are longer, with 172-280 amino acids and an extended C-terminal region, and normally encoded by the genes outside of the core T6SS locus (Lewis et al., 2019). Currently, the function of PAAR proteins in *A. baumannii* is not fully revealed yet. In this study, we investigated the role of a PAAR protein outside the core T6SS gene cluster, named TagP (T6SS-associated-gene PAAR), encoded by the gene ACX60_RS09070 outside the core T6SS locus of *A. baumannii* strain ATCC 17978.

## 2 Materials and methods

### 2.1 Bacterial strains, plasmids and cell lines

The bacterial strains and plasmids utilized in this study and their characteristics are outlined in **Table 1**. The bacterial *A. baumannii* ATCC 17978 strain utilized in this study was UN variant harboring AbaAL44 fragment (Wijers et al., 2021). The bacterial strains were consistently maintained in Luria-Bertani (LB) broth or agar. Carbenicillin at a concentration of 100 μg/mL, Kanamycin at 50 μg/mL, and Hygromycin at 300 µg/mL were added when necessary (Chen et al., 2017; Hua et al., 2021). Human Bronchial Epithelial Cell line (HBEpiC) and alveolar epithelial cell line A549 were cultured in Dulbecco’s modified Eagle’s medium (DMEM). The human macrophage cell line THP-1 was maintained in RPMI 1640 and supplemented with 10% fetal bovine serum and 1% penicillin-streptomycin. These strains and cells were supplied by the State Key Laboratory of Pathogen and Biosecurity in China. Plasmid pYMAb2-hyg was offered by the Key Laboratory of Microbial Technology and Bioinformatics of Zhejiang Province.

**Table 1:** Strains and plasmids used in this study.

Strain or plasmid	Characteristic (s)	Source or reference
Strains		
*A. baumannii* strain ATCC 17978	Reference strain	ATCC
17978-pAT02	*A. baumannii* 17978 carrying pAT02	(Tucker et al., 2014; Chen et al., 2017)
17978-pAT03	*A. baumannii* 17978 carrying pAT03	(Tucker et al., 2014; Chen et al., 2017)
Δ*tagP*::Kan^r^	*A. baumannii* 17978Δ*tagP*::Kan^r^	This study
Δ*tagP*::FRT	*A. baumannii* 17978Δ*tagP*::FRT	This study
Δ*tagP*::FRT-pAT03	*A. baumannii* 17978Δ*tagP*::FRT carrying pAT03	This study
Δ*tagP*::Com	*A. baumannii* 17978Δ*tagP*::FRT carrying pYMAb2 with RS09070	This study
Δ*tagP* -p	*A. baumannii* 17978Δ*tagP*::FRT carrying pYMAb2	This study
*E. coli* DH5α		Lab stock
*E. coli* MG1655		Lab stock
*E. coli* MJ109		Lab stock
Plasmids		
pKD4	Kan^r^	Lab stock
pYMAb2-hyg	Hyg^r^	(Hua et al., 2021)
pAT02	pMMB67EH with RecAb system	(Tucker et al., 2014; Chen et al., 2017)
pAT03	pMMB67EH with FLP recombinase	(Tucker et al., 2014; Chen et al., 2017)

### 2.2 Bioinformatics analysis and homology modeling

Genomes of *A. baumannii* ATCC 17978 were downloaded from the National Center for Biotechnology Information Public Database (https://ftp.ncbi.nlm.nih.gov) (GenBank: GCA_001077675.1) and verified by Sanger sequencing for the targeted genes. Amino Acid Sequence of PAAR-M protein ACX60_RS11625 and PAAR-C protein ACX60_RS09070 were aligned using multiple sequence alignment tools in https://toolkit.tuebingen.mpg.de/tools/ClustalΩservice.

### 2.3 Construction of *tagP* null and complement strains

The *tagP* gene ACX60_RS09070 of *A. baumannii* ATCC 17978 strain was deleted from the genome as previously described with minor modifications (Tucker et al., 2014). Briefly, a 2167bp DNA fragment was constructed using fusion PCR, which included 368 bases upstream and 600 bases downstream of the ACX60_RS09070 coding sequence (CDS). Primers 09070upf/r and 09070dnf/r were used to amplify the flanking regions, while primers Kanf/r were used to amplify the kanamycin resistance cassette from the pKD4 plasmid. The purified PCR product was then electroporated into *A. baumannii* carrying RecAb on pMMB67EH (pAT02 plasmid), which was maintained with carbenicillin. The kanamycin cassette in successful recombinants was deleted using pMMB67EH expressing the FLP recombinase (pAT03 plasmid). PCR was used to confirm the loss of the kanamycin cassette and the plasmids pAT02 and pAT03, resulting in the generation of the *tagP* null *A. baumannii* ATCC 17978Δ*tagP*::FRT (Δ*tagP*). A pair of primers 09070Rvf/r amplifying the ACX60_RS09070 and its 197bp upstream sequence with promoter were used to construct complement strains, and the pYMAb2-hyg plasmid was used as previously described to generate complement strain ATCC 17978Δ*tagP*::com (Δ*tagP*::com) and ATCC 17978Δ*tagP*-pYMAb2-hyg (Δ*tagP*-p) (Hua et al., 2021). PCR and sequencing were used to confirm the accuracy sequence of the *tap* gene. The primers adopted in this experiment were listed in the **Supplementary Table 1**.

### 2.4 Growth curve determination

The overnight cultures were 1:100 diluted with 60 mL of fresh LB in a 200 mL Erlenmeyer flask and maintained at 37°C with a shaking speed of 200 r/min. The absorbance at OD_600_ was automatically measured every 2 min for 36 h, and the results of the area under the curve from three trials were presented as the mean and standard deviation.

### 2.5 Biofilm assay

The biofilm formation ability of the strains was assessed using a previously established method (Tucker et al., 2014), with slight modifications that the 96-well polystyrene cell culture plate was incubated for 72 hours at 28°C without shaking, the bacterial fluid in twelve parallel repeating wells were determine the optical density (OD)_620_ to estimate the total cell biomass before being removed, and the remaining biofilms were rinsed and stained with 0.1% crystal violet according to the previously established method (Tucker et al., 2014), biofilm quantities were subjected to OD_590_/OD_620_ ratio.

### 2.6 Surface motility assays

The overnight cultures were 1:100 diluted with LB broth, and 1 μL of the bacterial suspension was placed at the center of the motility assay plate according to the reference (Chen et al., 2017). For swarming motility test, plates with 10 g/L tryptone, 5 g/L NaCl, and 0.3% Noble agar (Becton Dickinson, Sparks, MD, USA) were used; for twitching motility test, Mueller-Hinton (MH) medium with agar at a concentration of 0.4%.

### 2.7 Survivability in various simulated environmental conditions

LB medium containing a concentration gradient of 1.0, 2.0, and 3.0 M glucose (C_6_H_14_O_6_) was utilized to create a hyperosmotic environment; likewise, a concentration gradient of 10.0%, 15.0%, and 20.0% sodium chloride to form a high salinity environment; LB medium with pH values of 7.0, 6.0, 5.0, 4.0, and 3.5 to create different pH environments. 100 μL of the bacteria with an OD of 1.0 were added to 1.9 mL of the above medium for simulating at the setting time points. Pellets from 1 mL of the bacterial mixture were collected and washed twice with PBS before resuspended in the same volume of 0.01M pH7.4 PBS for plate counting. For the hydrogen peroxide (H_2_O_2_) resistance experiment, 200 mM and 20 mM H_2_O_2_ were added into PBS instead of LB medium. For the heat and cold temperature experiments, shake the bacteria in LB medium at 50°C for 30 min and at 4°C for 24 h, respectively. Further experiments with bacteria kept in pH 3.5 LB medium for 5, 15, and 30 minutes or PBS containing 20 mM H_2_O_2_ for 15, 30, 45, and 60 minutes were carried out to investigate the survival difference between the wild-type and the knockout strains.

### 2.8 Antimicrobial susceptibility testing

The minimal inhibitor concentrations (MICs) of common antibiotics (**Supplementary Table 2**) against *A. baumannii* were measured by the broth microdilution test card (GN13, bioMérieux, Lyons, France) according to the instructions of the manufacturer and the Clinical and Laboratory Standards Institute (CLSI, 2021; Humphries et al., 2021). The MICs of Gentamicin, Tigecycline and Colistin were further confirmed by the E-test (bioMérieux). *E. coli* ATCC 25922 was used as the quality control.

### 2.9 HBEpiC and A549 cell adhesion and invasion assays

Adhesion and invasion ability to HBEpiC and A549 cells were assayed. As recommended by Tang J et al (Tang et al., 2020), firstly, about 5 × 10^5^ cells per well were seeded in six-well plates in the culture medium of DMEM (Dulbecco’s modified Eagle’s medium) supplemented with 10% fetal bovine serum (Gibco) in a 37°C incubator with 5% CO_2_. For the infection experiments, cells adhesion and tightly connected growth until covered the bottom of the plate up to 80%, followed by bacterial infection with a multiplicity of infection (MOI) of 20. The infected cells were incubated for 2 h, then the infected monolayers were washed three times with PBS and lysed in 500 μL of 0.5% Triton X-100 before bacterial adhesion determination. To measure their invasion capability, cells in each well were supplemented with 200 μg/mL of gentamicin and incubated for 15 min; after that, dilutions of the lysates were plated on the LB agar for measurement. At each time point, two six-well plates were used for each strain, with three wells setting as controls without bacterial infection to exclude contamination by other bacteria during the experimental process. Three wells were used for adhesion experiments, three wells were used for invasion experiments, and the remaining one parallel well was used for Wright-Giemsa staining. Each strain was tested in triplicate.

### 2.10 Intramacrophage survival assay

Cell cultures were performed in accordance with the protocol in the references (Subashchandrabose et al., 2016; Sycz et al., 2021). Initially, 2 × 10^5^ cells were inoculated in each well of six-well plates and grew for 24 h before the test, where the culture medium was RPMI 1640 with 10% fetal bovine serum and 100 ng/mL PMA. After washing, the cells were co-cultured with the bacteria at a MOI of 50. Three technical replicates were exercised to assess the phagocytic ratio at 30 min and the survival ratio at 4, 8, and 12 h, and four identical plates were prepared for each assay at different time points (T_30_, T_240_, T_480_, and T_720_); dilutions of the bacteria were incubated with THP-1 cells for 30 min to allow for phagocytosis; 200 μg/mL Gentamicin was used to eliminate the extracellular bacteria. Phagocytes engulfing bacteria were collected at 15 min or 4, 8, and 12 h after the addition of gentamicin. At each time point, cells were lysed with 0.5% Triton X-100 and plated on the LB agar.

### 2.11 Serum survival assay

The serum resistance assay was determined following the procedure in previous research (Subashchandrabose et al., 2016). In brief, the overnight cultures were adjusted to a final concentration of 0.5 McFarl with PBS, and then 1:10 diluted to a final volume of 100 μL in either 90% serum or 90% heat-inactivated serum. Dilutions were plated at 0, 60, 120, and 180 min. The complement strains were cultured overnight in LB without antibiotic selection.

### 2.12 Preparation of the polyclonal antibody and western blotting

Expression of Hcp and its polyclonal antibody were prepared as previously described (Liu et al., 2016). Briefly, the *hcp* gene fragment from ATCC 17978 genomic DNA was amplified using HcpF/R primers (**Supplementary Table 1**), which introduced the *Nde*1 and *Bam*H1 restriction sites. The amplified fragments were then inserted into the pET-28a(+) expression vector with the addition of 0.5 mM IPTG to induce protein expression at 22°C. Following sequential chromatographic separation, Hcp was achieved with a high purity of > 95%, assessed by Coomassie Blue-stained SDS-PAGE. After that, the recombinant protein was diluted in sterile PBS and then mixed with Freund’s complete adjuvant for injection into the auricular vein of female New Zealand rabbits. The anti-Hcp polyclonal antibodies were used to detect native Hcp in the whole-cell lysates or supernatants by Western blotting with reference to the literatures (Shneider et al., 2013; Liu et al., 2016), and the protein RNAP was set as the control. Bacterial LB cultures were incubated at 28°C for 20 hours, followed by centrifugation to separate the bacterial cells and culture supernatant. 200 µL bacterial pellet was washed with 1 mL PBS for three times, then resuspended in 1 mL lysis buffer (6 mM NaCl, 1 mM NaH_2_PO4, 10 mM imidazole) containing a protease inhibitor at a ratio of 100:1 and sonicated to prepare the whole-cell lysates. Supernatant was filtered through a filter with 0.22 µm membrane. Then 5 µL of bacterial protein or 20 µL of supernatant was mixed with loading buffer to prepare WB samples.

### 2.13 Bacterial competition assay

The competition of bacteria of WT, Δ*tagP*, with *E. coli* MG1655 and MJ109 harboring pKD4 was assayed according to steps in the publication (Basler et al., 2013). Briefly, the killer and prey cells were mixed in equal proportions (1:1 ratio) and spotted onto the LB-agar plates. After incubating at 37°C for 4 h for the mixture with MJ109 and 6 h for the mixture with MG1655, the mixed cells were recovered in fresh LB and vigorously vortexed to release cells from the agar. The mixtures were then diluted and plated onto LB-agar plates with 50 μg/mL Kanamycin or 15 μg/mL Gentamicin for prey survival selection.

### 2.14 *Galleria melonella* killing assays

Following the protocol presented in the reference (Tang et al., 2020), *A. baumannii* bacteria were adjusted using PBS to match the turbidity of 0.5 McFarland. Twenty *G. melonella* of the same size were randomly selected to test the virulence of each bacterial strain. 20 μL of the suspension of each bacterium strain or PBS was injected into each *G. melonella*. After that, the *G. melonella* was preserved in a dark environment at 37°C for 4 days. The *G. melonella* was considered dead if it did not respond to gentle probing.

### 2.15 RNA-seq analysis

RNA sequencing analysis was conducted by Macro & Micro-test Bio-Tech Co., Ltd. (Beijing, China) (Hua et al., 2017). In brief, *A. baumannii* ATCC 17978 wild-type and the *tagP* null mutant strains were cultured overnight in LB broth at a temperature of 28°C. The bacterial cultures were then diluted 1:100 in fresh LB broth and incubated at 28°C for 4~5 hours and collected by centrifugation at 2,500 × *g* at 4°C for 15 minutes. RNA extraction was performed with the Pure Link™ RNA Mini Kit (Invitrogen, Carlsbad, CA, USA). The resulting sequencing reads were aligned to the ATCC 17978 genome using Bowtie2 v2.3.4.3 (Langmead and Salzberg, 2012). HTSeq v0.6.1 was employed to quantify the number of the reads mapped to each gene. Differential gene expression analysis was conducted using the DESeq R package (version 1.18.0) (Robinson et al., 2010). P-values were adjusted with the Benjamini and Hochberg method. Genes with an adjusted P-value < 0.05 and a log2 fold change > 1 were considered to be differentially expressed.

### 2.16 Proteomics analysis

LC-MS proteomic analysis was executed by PTM BioLab Co., Inc. (Hangzhou, China) (Song et al., 2020). Cultures were prepared as the same for RNA-Seq analysis. Three replicate cultures were used for each group. The resulting MS/MS data were processed with the MaxQuant engine (v.1.5.2.8, https://www.maxquant.org) searching against the proteomic database of ATCC 17978 in NCBI with GenBank accession number GCA_001077675.1. The parameters and the false discovery rate were set referred to the literature (Fei et al., 2022).

### 2.17 Quantitative reverse transcription PCR for Csu pili gene cluster

The biofilm formation and surface motility ability of *A. baumannii* was closely related to Csu pili (Chen et al., 2017; Moon et al., 2017). Since the biofilm formation and surface motility ability of the *tagP* null mutant strain was significantly reduced, we compared the expression of Csu pili genes between wild-type and the *tagP* null mutant strains cultured in LB broth at a temperature of 37°C by qRT-PCR assay. The RNAs were reverse transcribed into cDNA using SynScript III RT SuperMix (Tsingke Biotechnology Co., Ltd., Beijing, China). The genes of Csu pili gene cluster for qRT-PCR analysis were *csuAB*, *csuA*, *csuB*, *csuC*, *csuD* and *csuE.* The level of RNA polymerase β-subunit *(rpoB)* mRNA was used as a control. The primers for qRT-PCR were listed in the **Supplementary Table 4**.

### 2.18 Statistical analysis

Student’s t-test was employed to compare the mean value variances between the two groups. Chi-square and Fisher’s exact test were utilized for qualitative data analysis. Mean values and SEM from the three independent experiments were analyzed by one-way ANOVA, followed by Dunnett’s test for multiple comparisons. Survival curves were constructed using the Kaplan-Meier method and assessed using a log-rank (Mantel-Cox) test. All statistical analysis and plotting were based on R 4.3.0, and the Tidyverse and ggsurvey packages were used for data visualization (Xu et al., 2021). For all tests, a *P* value of < 0.05 was considered statistically significant.

### 2.19 Ethical statement

Animals in this study were treated following China’s guidelines for laboratory animal welfare and were conducted in accordance with the regulations outlined in laboratory animal permit No. SCXK (Jing) 2021-0006, obtained from Beijing Vital River Laboratory Animal Technology Co. Ltd. Human serum collection was approved by the ethical committee of Xiangya Hospital of Central South University (No. 202308645).

## 3 Results

### 3.1 Comparison of PAAR-M and PAAR-C

**Figure 1:**
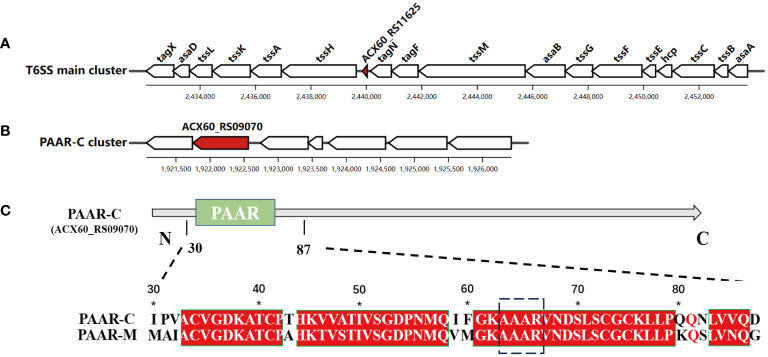
Two genes encoding PAAR proteins in *A*. *baumannii.* The genetic structure of PAAR-M (ACX60_RS11625) **(A)** and PAAR-C (ACX60_RS09070) loci **(B)**, the red color indicates their location. **(C)**. The sequence alignment of PAAR-M and PAAR-C reveals the identical PAAR domain in their N-terminal, while PAAR-C harbors an extended C-terminal region.

Among the genes encoding PAAR proteins in *A. baumannii*, ACX60_RS11625 (encoding PAAR-M) is situated in the main T6SS cluster (**Figure 1A**), while ACX60_RS09070 or *tagP* gene (encoding PAAR-C) resides in the auxiliary cluster (**Figure 1B**). Sequence alignment revealed that conserved sequence existed in their N-terminal (**Figure 1C**) while PAAR-M lacks the complex C-terminal structure possessed by PAAR-C, which suggests that the two PAAR proteins may have different biological functions.

### 3.2 Influence of *tagP* on growth kinetics of *A. baumannii*

**Figure 2:**
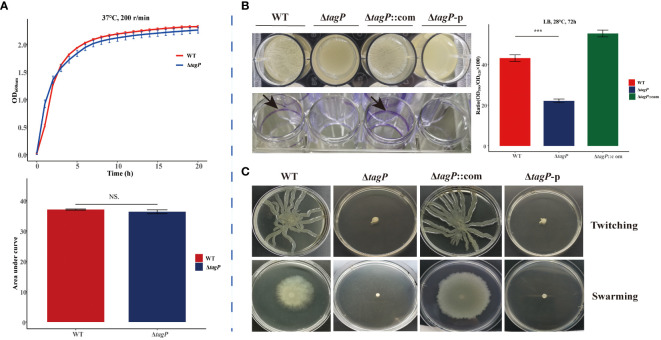
Growth kinetics, biofilm information and surface-related motility for *A*. *baumannii* ATCC17978, Δ*tagP*, Δ*tagP*::com and Δ*tagP*-p. **(A)** Growth curve and comparison of area under the curve. **(B)** Film on the surface of LB media or adhesion on the plate wells and quantified the biofilms using crystal violet analysis. **(C)** Motility assays with two kinds of plates (NS, non-significant; ***P < 0.001)..

The impact of the *tagP* gene on the growth of *A. baumannii* was investigated by comparing the *in vitro* growth rates of *A. baumannii* ATCC 17978 wild-type and the *tagP* null mutant strains. The growth kinetics of each strain in LB broth at 37°C was shown in **Figure 2A**. There was no significant difference in growth between the presence (wild-type) and absence (Δ*tagP*) of the *tagP* gene in *A. baumannii*.

### 3.3 Influence of *tagP* on biofilm formation of *A. baumannii*

After incubating at 28°C for 72 hours, biofilm formation between *A. baumannii* strains with and without *tagP* gene was observed and compared, including the film on the surface of LB liquid, the adhesion to polystyrene microtiter plate well, and the biofilm quantification using crystal violet staining. The results manifested that the wild-type strain formed clear biofilms and adhered to the microtiter plate wells, while the *tagP* null mutant strain did not form visible biofilms on the surface of the culture or the wells. Quantitative analysis using crystal violet confirmed significantly lower biofilm formation in the *tagP* null mutant strain compared to that in the wild-type strain (**Figure 2B**). Notably, biofilm formation was restored in the *tagP* complementation strain, in contrast, Δ*tagP*-p strain did not restore this phenotype. Therefore, we could conclude that *tagP* is crucial for *A. baumannii* to develop biofilm.

### 3.4 Influence of *tagP* on surface motility of *A. baumannii*

Although *A. baumannii* is commonly considered “non-motile”, its surface motility can be triggered under specific conditions and involved in its virulence. In order to investigate the impact of *tagP* on the surface motility of *A. baumannii*, the bacteria in the exponential phase were inoculated onto motility agar plates. Subsequent observation revealed that both twitching and swarming surface associated motility were significantly reduced in the *tagP* deletion mutant, whereas the complemented strains but not the Δ*tagP*-p strains exhibited similar surface motility to the wild-type ones (**Figure 2C**). These findings suggest that *tagP* participates in the surface motility of *A. baumannii*.

### 3.5 Influence of *tagP* on the survival ability of *A. baumannii* under simulated environmental conditions

**Figure 3:**
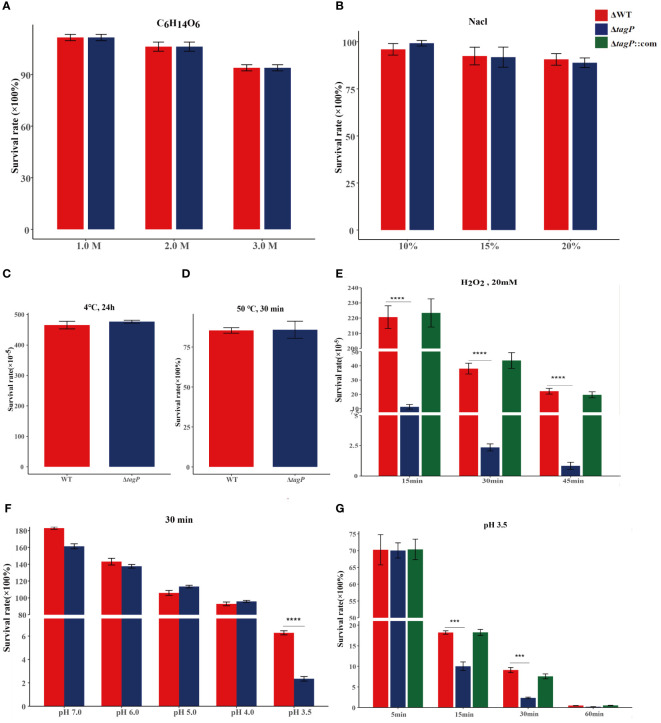
Survival of *A*. *baumannii* ATCC17978, Δ*tagP* in simulated high osmotic pressure with C_6_H_14_O_6_ **(A)**, high salt concentration with NaCl **(B)**, high temperature with 50°C **(C)**, low temperature with 4°C **(D)**, and oxidative stress with H_2_O_2_ **(E)**, acidic conditions with HCl **(F, G)**. Significant differences existed between wild-type and *tagP* null mutant strains to tolerant 20 mM H_2_O_2_ **(E)** and acid pH 3.5 **(G)** and totally restored by complemented strains. (***P < 0.001, ****P < 0.0001).

In various simulated extreme environments, the deletion of *tagP* was not found to significantly affect the adaptation of *A. baumannii* to changes in osmotic pressure (**Figure 3A**), salt concentration (**Figure 3B**), or temperature (**Figures 3C, D**); however, it showed lower tolerance than the wild-type strain to H_2_O_2_ stimulation (**Figure 3E**) or to acidic conditions with pH 3.5 (**Figures 3F, G**), while the complemented strain was able to restore tolerance in such conditions.

### 3.6 Influence of *tagP* on antimicrobial susceptibility

The MICs of ceftazidime (CAZ), cefepime (FEP), meropenem (MEM), imipenem (IMP), piperacillin/tazobactam (TZP), ticarcillin/clavulanic acid (TIM), piperacillin (PIP), levofloxacin (LVX), ciprofloxacin (CIP), gentamicin (GEN), tobramycin (TOB), amikacin (AMK), sulphamethoxazole (SXT), doxycycline (DOX), minocycline (MH), tigecycline (TGC) and colistin (COL) were measured. Of all results, there was no significant difference observed between wild-type and *tagP* null mutant strains (**Supplementary Table 2**).

### 3.7 Influence of *tagP* on adherence to HBEpiC and A549 cells

**Figure 4:**
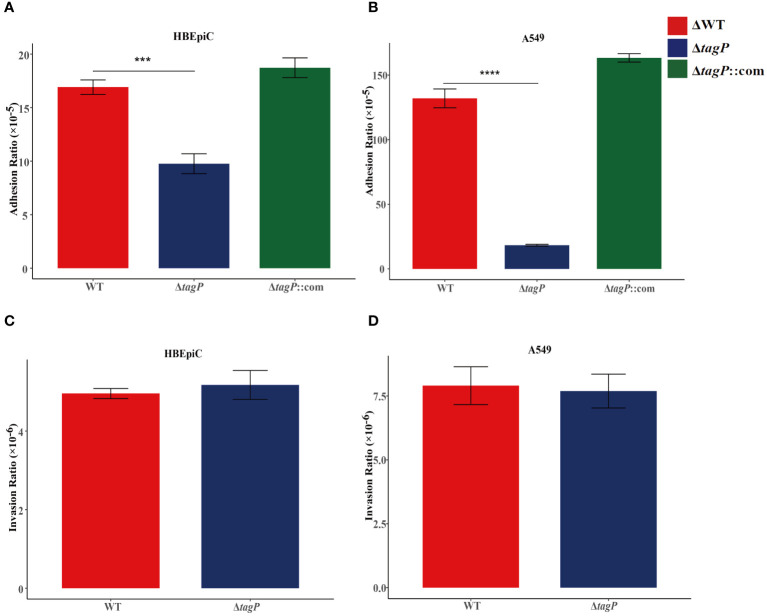
Adherence and invasion of *A*. *baumannii* ATCC17978, Δ*tagP* to HBEpiC and A549 cells. Significant differences in adhesion to either HBEpiC **(A)** or A549 **(B)** cells were observed, while no difference existed in the invasion of these cells **(C, D)**. ***P < 0.001, ****P < 0.0001.

HBEpiC bronchial epithelial cells and A549 alveolar epithelial cells were used to assess the *tagP* gene on the adhesion and invasion capacity of *A. baumannii*. It was observed that all of the *tagP* null mutant strains, the complemented strains, and the wild-type strains exhibited adhesion to HBEpiC and A549 cells; moreover, the *tagP* null mutant strains showed significantly lower adherence than the wild-type strain (**Figures 4A, B**), and the adherence was restored in the complemented strains. On the other side, all strains showed weak invasiveness toward either HBEpiC or A549 cells, and no significant differences were found among them (**Figures 4C, D**).

### 3.8 Influence of *tagP* on phagocytosis and survival in human monocyte-derived macrophage THP-1 cells

**Figure 5:**
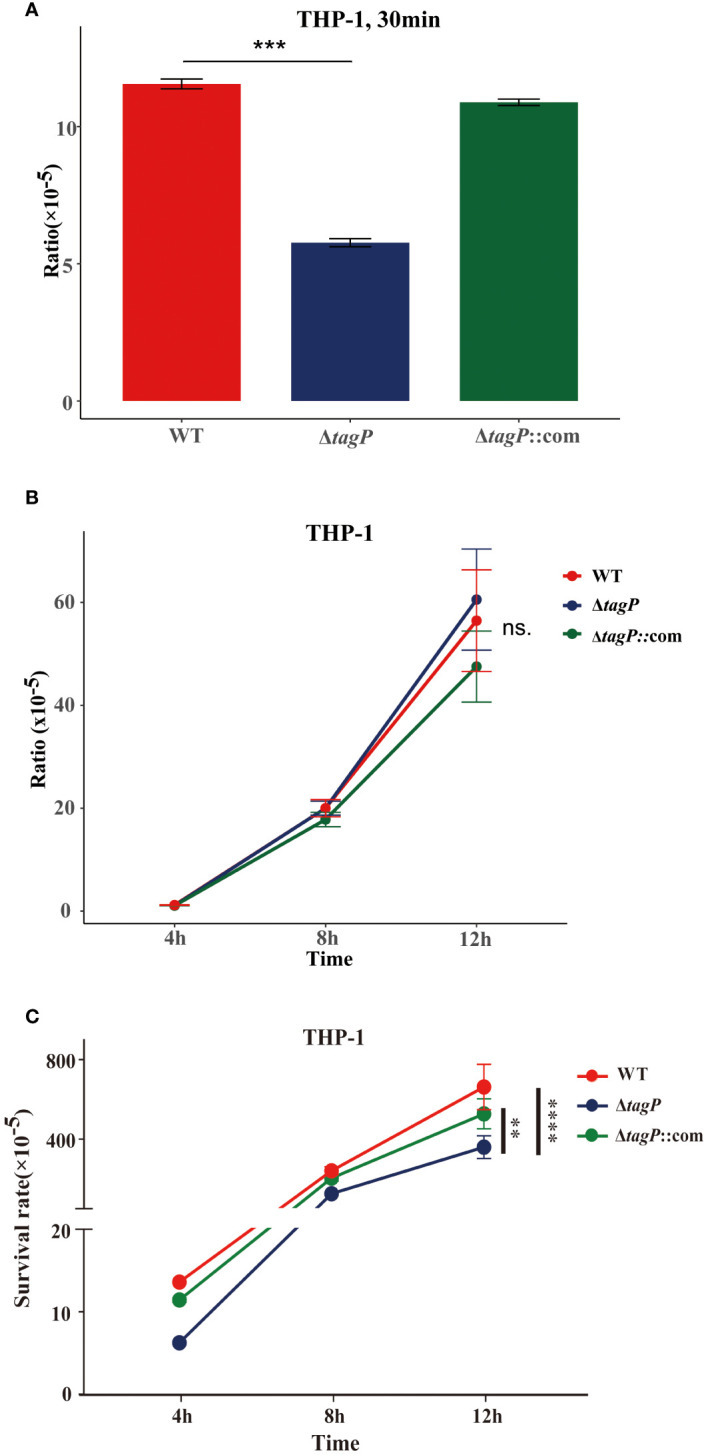
Phagocytosis and intramacrophage survival. **(A)**. Loss of *tagP* resulted in phagocytosis defects compared to the wild-type strain within THP-1 cells. **(B)**. The ratio of CFUs isolated from phagocytes at different time points to CFUs of phagocytic bacteria at 30 minutes. **(C)**. The ratio of CFUs isolated from cells at each time point relative to the initial input amount was determined. One-way ANOVA, followed by Dunnett’s test for multiple comparisons, was conducted on mean values and SEM from three independent experiments (ns, non-significant; **P < 0.01 ***P < 0.001 ****P < 0.0001).

The gentamicin protection assay was used to test whether *A. baumannii* mutants with impaired *in vivo* fitness resulted in compromised intramacrophage survival. The results revealed that within 30 min of phagocytosis, the *tagP* null mutant strains exhibited a significantly low phagocytosis rate compared to the wild-type strain (**Figure 5A**), while the complemented strains were restored from the phagocytosis defect. As some groups have shown that some strains of *Acinetobacter* can replicate inside macrophages (Sato et al., 2019; Sycz et al., 2021), both the wild-type and the null strains survived after 4, 8, and 12 h, and without significant difference in growth rate between them (**Figure 5B**). Thus, finally, a significantly higher load of the wild-type strains was isolated from macrophages (**Figure 5C**).

### 3.9 Influence of *tagP* on resistance to human serum

**Figure 6:**
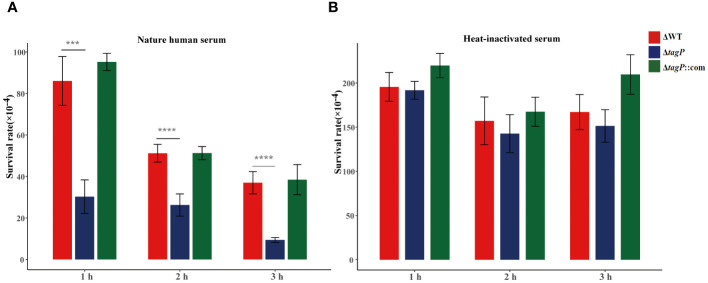
The *tagP* null mutant strain was more susceptible to complement mediated killing. **(A)**. Survival of wild-type and *tagP* null mutant strains in 90% human serum and heat-inactivated serum for various time points was assessed. The *tagP* null mutant strains showed decreased survival compared to the wild type in serum. However, heat inactivation of serum relieved the survival defect of the null strains; **(B)**. Mean rates of survival relative to the sizes of the inocula and SEM were calculated from three independent experiments. Statistical significance was determined using one-way ANOVA, followed by Dunnett’s test for multiple comparisons (***P < 0.001, ****P < 0.0001).

Heat inactivation is a common method to deactivate complement, the main factor that kills bacteria in serum. Investigation of the survival difference between the wild-type and the *tagP* null mutant strains in the natural human serum or the heat-inactivated serum proved that the wild-type strain had a significant reduction in viability in natural serum but insignificant difference in the heat-inactivated serum after 60, 120, and 180 min upon exposure (**Figure 6**), indicating that complements may involve killing the bacteria in serum. Obviously, compared to the parental strain, the *tagP* null mutant strains appeared to be more susceptible to natural serum with the extension of exposure time (**Figure 6A**). However, such survival reduction was not discovered in the heat-inactivated serum (**Figure 6B**), suggesting that the *tagP* null mutant strain is highly susceptible to complement mediated killing and the *tagP* gene may specifically contribute to the complement resistance.

### 3.10 Influence of *tagP* on the secretion of Hcp

**Figure 7:**
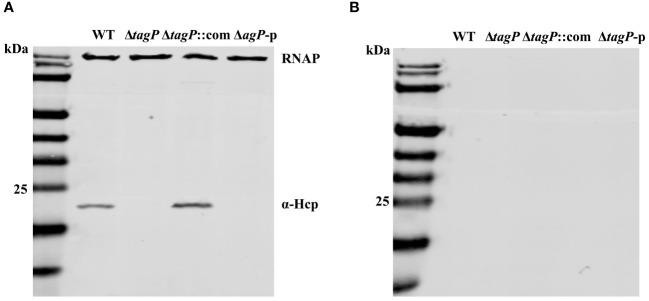
Hcp expression in the whole cells **(A)** and supernatants **(B)** of *A*. *baumannii* ATCC 17978 wild-type, Δ*tagP*, Δ*tagP*::com and Δ*tagP*-p strains.

We assessed whether TagP affects the expression and secretion of Hcp in *A. baumannii*. As shown in **Figure 7**, Hcp protein was identified in the whole-cell lysates of the wild type strain and the complemented strain, but not in the *tagP* null mutant and Δ*tagP*-p strain. Western blotting failed to detect Hcp protein in the culture supernatants of all strains including the wild type strain. Weber et al. discovered that two regulators encoded by the plasmid pAB3 can suppress the expression and secretion of Hcp in ATCC 17978 (Weber et al., 2015). Our results are consistent with their findings, and further imply that the presence of *tagP* gene is critical for the expression of Hcp in *A. baumannii*.

### 3.11 Influence of *tagP* on the competitive killing of *E. coli*

**Figure 8:**
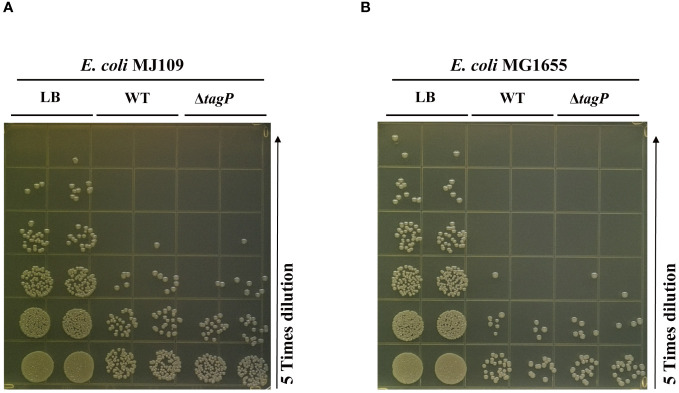
Bacterial competition assay assessing the competitive killing ability of *A*. *baumannii* ATCC 17978 wild-type, *tagP* null mutant strains against *E*. *coli* MJ109 **(A)** and *E*. *coli* MG1655 **(B)**. The presence or absence of *tagP* did not affect the ability of *A*. *baumannii* to competitively kill prokaryotic organisms.

To test whether *tagP* influences the killing activities against the *E. coli* prey, we used MJ109 and MG 1655 as prey cells to conduct multiple repeated experiments, and found out that the deletion of *tagP* did not lead to significant changes in killing efficiency (**Figure 8**). Weber et al. proved that the presence of pAB3 plasmid suppressed the bacteria-killing ability of ATCC17978, the pAB3^-^ ATCC17978 only showed residual kill ability to *E. coli* (Weber et al., 2015). In our assays, the wild type strain and the *tagP* null mutant failed to kill the high titers of *E. coli*. Our results demonstrated that strains absence of *tagP* still possess the bacterial antagonistic ability, implying that multiple effectors might be involved in the prokaryotic competitive killing in *A. baumannii* other than Hcp or T6SS.

### 3.12 Influence of *tagP* on virulence of *A. baumannii* infection in the *G. mellonella* model

**Figure 9:**
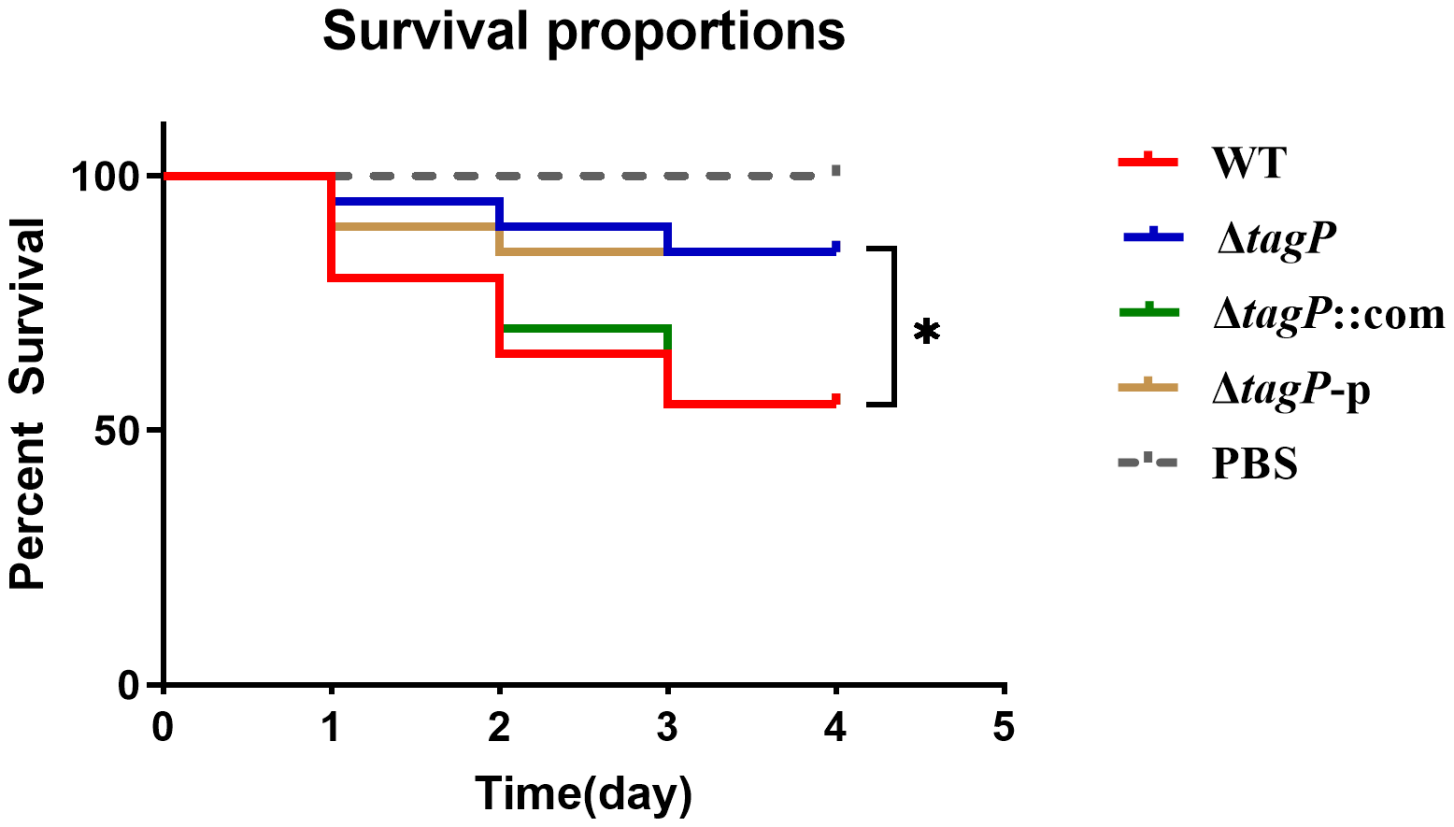
Survival curves *of G. mellonella* infected with *A. baumannii* strains. *tagP* harboring *A. baumannii* caused significantly higher mortality compared to the *tagP* null mutant strains (*P < 0.05).

*G. mellonella* is often used to study host-pathogen interactions. The *G. mellonella* model is considered reliable for studying the virulence and pathogenesis of *A. baumannii* (Peleg et al., 2009). As shown in **Figure 9**, significant differences (*P* < 0.05) in virulence targeting *G. mellonella* were uncovered between the wild-type and the *tagP* null mutant strains, where the *G. mellonella* infected with wild-type strain exhibited a mean death rate of 55%, while less than 20% when infected with the *tagP* null mutant strains.

### 3.13 Quantitative transcriptomic and proteomic analyses

A total of 501 upregulated and 213 downregulated TagP-dependent genes were identified by RNA-seq analysis satisfying the criteria of log2 Fold change > 1 and padj < 0.05 (**Supplementary Table 3**, tab A and B, respectively, and **Supplementary Table 5**). Based on the Kyoto Encyclopedia of Genes and Genomes (KEGG) classifications, genes were broadly distributed among the KEGG categories (**Supplementary Figure 1**). Nevertheless, the majority of them correlative to *tagP* fell into the “microbial metabolism in diverse environment category”, where the upregulated genes were mainly gathered in the amino acid metabolism pathway, while the downregulated clustered in the glycometabolism and ribosome. Simultaneously, quantitative proteomic analysis determined a total of 108 upregulated and 104 downregulated TagP-dependent proteins as presented in **Supplementary Table 3**, tab C and D, respectively, including 61 upregulated and 59 downregulated proteins fulfilling the criteria of log2 Ratio > 1 and *P* value < 0.05. In addition, these differentially expressed genes went through enrichment analysis to further determine their biological functions (**Supplementary Figure 2**).

Genes within the T6SS main cluster that exhibited significant changes in transcription levels were shown in **Table 2**; significant expression changes ranged from *tssH* (ACX60_RS11620) to *asaA* (ACX60_RS11680), indicating a closely functional relationship between *tagP* and the T6SS genes; TssB, TssC, Hcp, and AsaA exhibited consistent changes at the protein level.

**Table 2:** T6SS genes with significant changes in expression after *tagP* gene deletion.

gene_locus	gene_name	Log2.Fold_change based on RNA-seq (*p*adj)	Δ*tagP*/WT Ratio based on proteome *(p* value)
ACX60_RS11680	*asaA*	-5.46 (0)	0.01 (0)
ACX60_RS11675	*tssB*	-5.66 (0)	0.04 (3.94E-06)
ACX60_RS11670	*tssC*	-4.8 (0)	0.23 (9.30E-05)
ACX60_RS11665	*hcp*	-3.95 (0)	0.17(0.04)
ACX60_RS11660	*tssE*	-2.97 (3.04E-75)	–^a^
ACX60_RS11640	*tssM*	-1.86 (7.90E-70)	0.68 (–^b^)
ACX60_RS11645	*asaB*	-1.69 (4.04E-19)	–
ACX60_RS11655	*tssF*	-1.46 (3.67E-31)	–
ACX60_RS11650	*tssG*	-1.2 (1.19E-07)	–
ACX60_RS11635	*tagF*	-1.18 (2.28E-08)	–
ACX60_RS11630	*tagN*	-1.17 (1.47E-12)	–
ACX60_RS11620	*tssH*	-1.02 (1.11E-28)	1.04 (–)

^a^No reads or peptides from the protein were detected based on 4D label-free quantitative proteomic assay. ^b^Reads or peptides did not detect from some of the three duplicate samples, making it unsuitable for calculating p-values.

Twenty-six genes outside the T6SS main cluster were found to be consistently upregulated at both the transcription and the protein level, and 31 genes were downregulated, as presented in **Tables 3**, **4**. Notably, H-NS was significantly upregulated in the *tagP* null mutant. Several membrane proteins, including the OmpA family protein, Trimeric autotransporter adhesin Ata, type 1 fimbrial protein, surface antigen protein SurA1, and a gene cluster (ACX60_17480-17515) responsible for lipopeptide encoding, were significantly downregulated.

**Table 3:** TagP-dependent upregulated genes out of T6SS main cluster with expressions log2 Fold change and log2 ratio > 1 and *p*< 0.05 in *A baumannii* analyzed using RNA -seq and 4D label-free quantitative proteome.

gene_id	gene_name	transcriptome^a^	proteome^b^	gene_description
ACX60_RS16750		2.91	7.88	H-NS histone family protein
ACX60_RS00325	*hppD*	2.90	1.91	4-hydroxyphenylpyruvate dioxygenase
ACX60_RS00340	*maiA*	2.20	1.82	Maleylacetoacetate isomerase
ACX60_RS00335		2.16	2.78	VOC family protein
ACX60_RS17550		2.14	1.91	AMP-binding protein
ACX60_RS00345	*fahA*	2.14	1.73	Fumarylacetoacetase
ACX60_RS08490		2.10	1.59	MFS transporter
ACX60_RS12155	*benB*	2.01	1.21	Benzoate 1%2C2-dioxygenase small subunit
ACX60_RS06830		2.00	1.44	AMP-binding protein
ACX60_RS12800		1.96	1.37	Feruloyl-CoA synthase
ACX60_RS11265		1.95	1.88	AMP-binding protein
ACX60_RS08485		1.76	2.25	OprD family outer membrane porin
ACX60_RS13395		1.75	1.03	SRPBCC domain-containing protein
ACX60_RS00945		1.75	1.45	DUF485 domain-containing protein
ACX60_RS03155		1.73	1.68	AraC family transcriptional regulator
ACX60_RS02455		1.69	1.12	GFA family protein
ACX60_RS12150	*benA*	1.58	1.45	Benzoate 1%2C2-dioxygenase large subunit
ACX60_RS05720	*xdhA*	1.54	1.41	Xanthine dehydrogenase small subunit
ACX60_RS13830		1.47	1.4	Gamma-glutamyltransferase family protein
ACX60_RS04550		1.38	1.45	CSLREA domain-containing protein
ACX60_RS17875		1.33	1.5	acyl-CoA desaturase
ACX60_RS12160	*benC*	1.25	1.17	Benzoate 1%2C2-dioxygenase electron transfer component BenC
ACX60_RS11285		1.17	1.16	Enoyl-CoA hydratase/isomerase family protein
ACX60_RS08735		1.14	1.14	Long-chain-fatty-acid–CoA ligase
ACX60_RS18570		1.04	2.27	DNA topoisomerase
ACX60_RS08600		1.01	2.71	Muconate/chloromuconate family cycloisomerase

The table summarized the data from three independent biological replicates of wild type (WT) A baumannii ATCC 17978 and the tagP null mutants, ^a^log2 Fold Change (mutants/wt) > 1 and p<0.05 based on RNA-seq analysis; ^b^log2 ratio (mutants/wt) > 1 and p<0.05 based on 4D label-free quantitative proteomic analysis. No reads or peptides from TagP were detected in the tagP null mutants. For spectrometry analysis, the ratio was determined by imputing correlating intensities around the detection limit.

**Table 4:** TagP-dependent downregulated genes out of T6SS main cluster with expressions log2 Fold change and log2 ratio< -1 and *p*<0.05 in *A baumannii* analyzed using RNA -seq and 4D label-free quantitative proteome.

gene-id	gene_name	Transcriptome^a^	Proteome^b^	gene_description
ACX60_RS20025		-5.86	-4.13	KGG domain-containing protein
ACX60_RS17510		-5.59	-1.81	Fatty acyl-AMP ligase
ACX60_RS17505		-5.52	-2.84	Acyl-CoA dehydrogenase
ACX60_RS17490		-5.27	-4.68	Outer membrane lipoprotein-sorting protein
ACX60_RS17495		-5.22	-2.18	Non-ribosomal peptide synthetase
ACX60_RS06400		-4.81	-2.36	DUF2171 domain-containing protein
ACX60_RS17480		-4.6	-2.75	Alpha/beta fold hydrolase
ACX60_RS11355	*alr*	-4.26	-6.06	Alanine racemase
ACX60_RS11220	*surA1*	-3.78	-2.15	Hypothetical protein
ACX60_RS11200	*katE*	-3.24	-2.05	Catalase HPII
ACX60_RS14350		-3.06	-1.78	Trehalose-6-phosphate synthase
ACX60_RS11995		-2.93	-4.54	Hypothetical protein
ACX60_RS10570		-2.65	-1.87	type 1 fimbrial protein
ACX60_RS09365		-2.2	-1.16	NAD(P)/FAD-dependent oxidoreductase
ACX60_RS14345	*otsB*	-2.01	-1.38	Trehalose-phosphatase
ACX60_RS12000		-1.96	-2.71	DUF4142 domain-containing protein
ACX60_RS11685		-1.91	-1.66	SUMF1/EgtB/PvdO family nonheme iron enzyme
ACX60_RS11100		-1.85	-2.57	Family 2A encapsulin nanocompartment shell protein
ACX60_RS11765		-1.8	-1.67	Glutathione-dependent formaldehyde dehydrogenase
ACX60_RS13220	*ata*	-1.76	-2.59	Trimeric autotransporter adhesin Ata
ACX60_RS11560		-1.6	-2.83	Epoxyqueuosine reductase QueH
ACX60_RS09395	*lpdA*	-1.53	-1.24	Dihydrolipoyl dehydrogenase
ACX60_RS05025		-1.47	-2.19	Hypothetical protein
ACX60_RS13215		-1.44	-3.01	OmpA family protein
ACX60_RS01085		-1.37	-1.69	Hypothetical protein
ACX60_RS05020		-1.33	-1.6	Hsp70 family protein
ACX60_RS04305		-1.28	-3.22	TIR domain-containing protein
ACX60_RS05010		-1.2	-1.05	PaaI family thioesterase
ACX60_RS12970		-1.09	-3.16	FAD-dependent monooxygenase
ACX60_RS03945		-1.07	-1.88	Hypothetical protein
ACX60_RS13925		-1.05	-1.5	Bacteriohemerythrin

^a^log2 Fold Change (mutants/wt) < -1 and p<0.05 based on RNA-seq analysis; ^b^log2 ratio (mutants/wt) < -1 and p<0.05 based on 4D label-free quantitative proteomic analysis.

### 3.14 Influence of *tagP* on expression of Csu pili gene cluster

To investigate whether Tap affects the expression of Csu pili genes, which were closely related to the biofilm formation and motility ability of *A. baumannii*, we utilized qRT-PCR to detect the expression of Csu genes in *A. baumannii* with or without TagP under dynamic culture conditions. The results (**Supplementary Figure 3**) showed that the Csu gene cluster, including CsuAB to CsuE, was significant downregulation in the *tagP* null mutant strains.

## 4 Discussion

*A. baumannii* is an important opportunistic pathogen in hospital infections, causing infectious diseases such as septicemia, pneumonia, meningitis, urinary tract infections, and wound infections (Fournier and Richet, 2006; Munoz-Price and Weinstein, 2008). However, as a notorious human pathogen, its molecular mechanism of infection has not been fully understood.

The PAAR proteins have been found to have various functions, including being a core structural component of T6SS (Shneider et al., 2013; Cianfanelli et al., 2016), an effector itself (Whitney et al., 2015), an effector carrier (Burkinshaw et al., 2018), or a selection controller for specific VgrG secretion (Cianfanelli et al., 2016; Wood et al., 2019b). Few papers delved into the functions of PAAR proteins other than the T6SS system and prokaryotic antagonisms. A bioinformatics analysis suggested that the PAAR genes might promote the environmental adaptation of bacteria, which still awaits experimental confirmation (Zheng et al., 2021). PAAR might get involved in the stress responses (ROS, temperature, and pH) and host adaptations of bacteria, as part of T6SS (Yu et al., 2021). The presence of multiple PAAR proteins with non-conservative C-extension regions in most bacterial species makes it challenging to determine their precise roles. In our study, we selected the *A. baumannii* strain ATCC 17978, which has a unique core T6SS cluster and a distinct PAAR protein outside the core region, to investigate the role of the PAAR protein outside the main T6SS cluster.We investigated the role of TagP in the function of T6SS in *A. baumannii* by Hcp detection and the bacterial competition assay. Through Western blotting and quantitative transcriptomic and proteomic analyses, our results disclosed that Hcp expression was significantly downregulated. Our transcriptome analysis revealed that the deletion of *tagP* led to a significant downregulation of a number of genes in the core T6SS gene cluster, including *tssB*, *tssC*, *tssE*, *tssM*, and *hcp*. A consistent reduction in either transcription or protein level was observed for TssB, TssC, and Hcp. TssB and TssC are components of the TssB-TssC complex that constitutes the sheath of the tail-tube in the T6SS assembly. For competition killing assay, the *tagP* null mutant strain retained its ability to kill *E coli*, which indicating prokaryotic antagonism of *A. baumannii* may be a cellular function involving multiple effectors, and stable expression of other effectors may rescue competitive damage caused by reduced Hcp expression. The specific aspects in which TagP influences the T6SS function of *A. baumannii* deserve further exploratory research.

We also investigated the impact of TagP on the adaptability to the environment and its pathogenicity towards the host of *A. baumannii*. Through exploration of various phenotypes, we found that the deletion of TagP leads to the defect of surface motility, adhesion, and the biofilm formation of *A. baumannii*, as well as its tolerance to acid and H_2_O_2_. As for the pathogenicity towards the host, the deletion of TagP leads to decreased adhesion to the host respiratory epithelial cells, low phagocytosis rate, as well as lower resistance to serum complement killing. We observed consistent phenotypic changes implying decreased bacterial adhesive ability in the *tagP* null strain in this study. The quantity of bacteria attaching to the culture plate surface decreases, contributing to a reduction in biofilm formation on the solid surface. Meanwhile, the *tagP* deletion results in a decrease in the number of bacteria adhering to respiratory epithelial cells in the gentamicin protection assay. Additionally, a low phagocytosis rate could be a result of fewer cells adhering to macrophages and other factors. Previous investigations have shown a close association between T6SS and bacterial adhesion, as well as biofilm formation. For example, Liu et al. demonstrated that T6SS deletion mutants in *Citrobacter freundii* influenced flagellar gene expression and secretion, impacting adhesion and cytotoxicity to host cells (Liu et al., 2015). In *Klebsiella pneumoniae*, T6SS was found to be linked to antibiotic resistance and biofilm formation (Mohamed et al., 2023). Bacterial adhesion plays a critical role in biofilm formation, serving as a necessary factor in the initial stages of biofilm development. Drugs or biomaterials were used to disrupt adhesion mechanisms, reducing initial bacterial attachment or implementing surface modifications to prevent biofilm formation (Qiu et al., 2019; Xu and Siedlecki, 2022). Although we observed a significant inhibition of T6SS expression due to the absence of *tagP*, it is still unclear to us whether the suppression of T6SS leads to the decreased adhesive ability of *tagP* null mutant in a direct or indirect way.

The surface motility of bacteria is influenced by many factors, including the rotation of rigid helical filaments, assembly-disassembly of pilis, biosurfactants, and quorum sensing. Bacteria utilize type IV pili for twitching motility on solid and semisolid surfaces, which can retract autonomously or in a synchronized manner, facilitating controlled movements (Harding et al., 2013; Corral et al., 2021). Biosurfactants are utilized for surface motility by reducing interfacial surface tension and increasing the solubility of hydrophobic compounds (Sharma et al., 2021). Quorum sensing is involved in the surface motility and biofilm formation in various bacteria (Weiss et al., 2008; Mayer et al., 2020). Although no significant changes were found in the expression of type IV pili and quorum sensing system genes in our transcriptome and proteome analysis, we did observe a significant inhibition in the expression of the *csu* pili gene cluster in *tagP* null mutant strains. Other than *csu* genes, we also observed a significant inhibition in the expression of a large gene cluster (ACX60_RS17480-17515) in *tagP* null mutant strains. This gene cluster is likely to encode lipopeptides and might produce a biosurfactant involved in surface motility.

In addition, genes other than T6SS with significant expression and close relevance to the phenotypes were well identified in our transcriptomic and quantitative proteomic analyses, such as the downregulation of OmpA, Ata, type 1 fimbrial protein, and Catalase. The role of outer membrane protein OmpA in the virulence of *A. baumannii* has been thoroughly investigated, revealing its adherence to eukaryotic cell surfaces and cellular invasion (Del Mar Tomas et al., 2005; Choi et al., 2008; Jin et al., 2011). One of the most commonly observed protein structures decorating the outer surface of pathogens is type 1 fimbrial. It is vital in the adherence to gram-negative pathogens (Ramezanalizadeh et al., 2020). Catalase, which is responsible for resisting intracellular reactive oxygen species (ROS), has been proven to be dependent on H-NS encoded plasmids for expression in ATCC 17978 or a modern *A. baumannii* urinary isolate UPAB1 (Benomar et al., 2021; Squire et al., 2022). Ata from *A. baumannii* has been shown to be essential in bacterial adherence and virulence (Bentancor et al., 2012). Ata (ACX60_RS13220) and OmpA-like protein (ACX60_RS13215) have been found to be co-transcribed, and both are regulated by H-NS (Eijkelkamp et al., 2013).

Notably, the deletion of *tagP* led to a significant upregulation of the H-NS (ACX60_RS16750) (Ratio of mu/wt based on quantitative proteome = 235.9, log2 Ratio = 7.9). H-NS is a global regulator that plays a critical role in orchestrating the bacterial life cycle (Ayala et al., 2017). It exerts negative control over numerous genes, thereby governing the overall virulence network of the bacteria, which has gained significant attention. Several studies have demonstrated that H-NS can regulate various phenotypes in *A. baumannii*. Rodgers et al. observed that the quorum network genes were up-regulated in *hns* mutant *A. baumannii* strains, and led to a broader role in the regulation of motility and surface sensing behaviors (Rodgers et al., 2021). Eijkelkamp et al. also discovered that the loss of regulatory control by H-NS led to differences in surface motility in *A. baumannii* (Eijkelkamp et al., 2013). What’s more, by inactivating H-NS, Eijkelkamp et al. found an increase in the expression of genes of Ata, T6SS, type I pilus cluster, alanine racemase (Alr), and the quorum sensing system (Eijkelkamp et al., 2013). Similarly, the expression of Ata, T6SS, type I pilus, and Alr were suppressed in the *tagP* null mutant in this study. Alr is an important protein that affects the motility of *A. baumannii* by chemoreception regulating (Irazoki et al., 2023). Ata, T6SS, and type I pilus, as we discussed above, are all crucial factors involved in bacterial adherence and biofilm formation. Combining all phenotypes and the significant overexpression of H-NS in *tagP* null mutant, it suggests that the presence of TagP might suppress the expression of H-NS and in turn, maintain various functions of *A baumannii*.

Although our findings indicated that *tagP* is a factor that affects the expression of H-NS, the specific regulatory mechanism- has not been clarified. Most existing investigations on H-NS focused on its downstream regulatory action (Ayala et al., 2017), suggesting the need for more attention to its upstream regulation mechanisms. Furthermore, among the genes that showed significant concurrent downregulation in both transcriptomic and proteomic analyses, some are functionally linked to the aforementioned phenotypes. However, to date, we have found few studies providing evidence of the critical role of H-NS in the regulation of their expression, for example, the surface antigen protein 1 (SurA1), which is closely related to the adherence of *A. baumannii* to eukaryotic cells (Liu et al., 2016). For other significantly downregulated proteins that their functions are unknown or have not been well characterized, our findings suggested their potential connection with the observed phenotypic changes in *A. baumannii* and indicated that they may serve as promising candidate virulence factors.

On the other hand, along with the increased expression of H-NS, there were other genes showing modest increases in expression of transcriptional and protein levels (2- to 3-fold) in the *tagP* null mutant, including multiple AMP-binding proteins related to energy metabolism, the oxidoreductase-related benABC system related to bacterial survival and growth within macrophages, and the long-chain-fatty-acid, which is associated with the inhibition of functionality of T6SS (Shao et al., 2022).

In summary, the data of this study demonstrated that TagP in *A. baumannii* was involved in a complex network of functional regulation. It enriches our understanding of the biofunction of PAAR proteins, which may contribute to the expansion of T6SS function but is not limited to T6SS. This research highlights the significance of TagP in the unique modulation of fitness and virulence control in *A. baumannii*.

## Data availability statement

The datasets presented in this study can be found in online repositories. The names of the repository/repositories and accession number(s) can be found below: https://nmdc.cn/resource/genomics/sra/detail/NMDC40042157, NMDC40042157; https://nmdc.cn/resource/genomics/sra/detail/NMDC40042158, NMDC40042158; https://nmdc.cn/resource/genomics/sra/detail/NMDC40042159, NMDC40042159; https://nmdc.cn/resource/genomics/sra/detail/NMDC40042160, NMDC40042160; https://nmdc.cn/resource/genomics/sra/detail/NMDC40042161, NMDC40042161; https://nmdc.cn/resource/genomics/sra/detail/NMDC40042162, NMDC40042162; https://nmdc.cn/resource/genomics/miscellan/detail/NMDC0000038, NMDC0000038; https://nmdc.cn/resource/genomics/miscellan/detail/NMDC0000039, NMDC0000039; https://nmdc.cn/resource/genomics/miscellan/detail/NMDC0000040, NMDC0000040; https://nmdc.cn/resource/genomics/miscellan/detail/NMDC0000041, NMDC0000041; https://nmdc.cn/resource/genomics/miscellan/detail/NMDC0000042, NMDC0000042; https://nmdc.cn/resource/genomics/miscellan/detail/NMDC0000043, NMDC0000043; https://nmdc.cn/resource/genomics/miscellan/detail/NMDC0000044, NMDC0000044.

## Ethics statement

The studies involving humans were approved by the ethical committee of Xiangya Hospital of Central South University (No. 202308645). The studies were conducted in accordance with the local legislation and institutional requirements. The participants provided their written informed consent to participate in this study. The animal studies were approved by Animals in this study were treated following China’s guidelines for laboratory animal welfare and were conducted in accordance with the regulations outlined in laboratory animal permit no. SCXK (Jing) 2021-0006, obtained from Beijing Vital River Laboratory Animal Technology Co. Ltd. The studies were conducted in accordance with the local legislation and institutional requirements. Written informed consent was obtained from the owners for the participation of their animals in this study.

## Author contributions

YL: Funding acquisition, Investigation, Writing – original draft. YC: Investigation, Writing – original draft. KS: Writing – original draft, Methodology. LS: Methodology, Writing – original draft, Investigation. LX: Methodology, Writing – original draft, Validation. JJ: Methodology, Validation, Writing – original draft. YZ: Methodology, Writing – original draft. YY: Writing – original draft, Software, Visualization. SZ: Software, Writing – original draft, Data curation. WY: Writing – original draft, Investigation, Methodology. SW: Writing – original draft, Resources. ZD: Resources, Formal analysis, Writing – review & editing. RY: Writing – review & editing, Conceptualization, Supervision. BY: Writing – review & editing, Project administration, Resources. YS: Project administration, Writing – review & editing, Conceptualization, Supervision.

## Supplementary material

Supplementary Table 1 The primers used in this study.

Supplementary Table 2 The minimal inhibitor concentrations (MICs) of the ATCC 17978 wild-type and *tagP* null mutant strains to common antibiotics.

Supplementary Table 3 TagP-dependent regulated genes. TagP-dependent up or down-regulated genes based on RNA-seq were listed in tab A and B, respectively. Up or down-regulated proteins based on 4D free LC-MS were listed in tabs C and D.

Supplementary Table 4 Primers used for detecting the expression of Csu pili genes by qRT-PCR.

Supplementary Table 5 RNA-seq of genes of the accessory cluster present in Ab 17978 UN strain.

Supplementary Table 6 qRT–PCR assays for detecting the mRNA in the ATCC 17978 wild-type and the *tagP* null mutant strains.

Supplementary Figure 1 KEGG pathway enrichment analysis of TagP-dependent regulated genes based on RNA-seq. KEGG pathway enrichment analysis was performed with a significance threshold of padj < 0.05. The top 20 significantly enriched KEGG pathways of upregulated genes **(A)** or downregulated genes **(B)** were selected and depicted in a scatter plot. The x-axis represents the ratio of differentially expressed genes annotated to the KEGG pathway to the total number of differentially expressed genes. The y-axis represents the KEGG pathway, with color shading ranging from purple to red. The intensifying red color indicates a more significant enrichment. Additionally, larger dots represent a higher number of genes enriched in the pathway.

Supplementary Figure 2 Cluster analysis of TagP-dependent regulated proteins based on quantitative proteome. Split the proteins into four groups (Q1 to Q4) based on fold change. Perform GO, KEGG, Reactome, Protein domain, and Wiki Pathways enrichment and functional clustering for each group. Apply hierarchical clustering to group together related functions in different Q groups, using Fisher’s exact test *P* value as criteria. Generate a heatmap with Q groups displayed horizontally and enriched functions displayed vertically. The intensity of the color blocks represents the significance of enrichment, with blue indicating high significance and blue-white indicating low significance. * denote *P* < 0.05, ** for *P* < 0.01, and *** for *P* < 0.001.

Supplementary Figure 3 qRT–PCR assays for detecting the mRNA in the ATCC 17978 wild-type strains and the *tagP* null mutant strains. The results shown were the means ± S.D. (n = 3) relative to the wild-type strains results. *rpoB* was used as the internal control. *, P < 0.05 and **, P < 0.01 compared with wild-type strains based on Student’s t-test.
