# Supplementary figures and images for: TagP, a PAAR-domain containing protein, plays roles in the fitness and virulence of Acinetobacter baumannii
Source: Front Cell Infect Microbiol. 2024 Jul 18;14:1379106. doi: 10.3389/fcimb.2024.1379106 (PMC11348943; doi:10.3389/fcimb.2024.1379106)

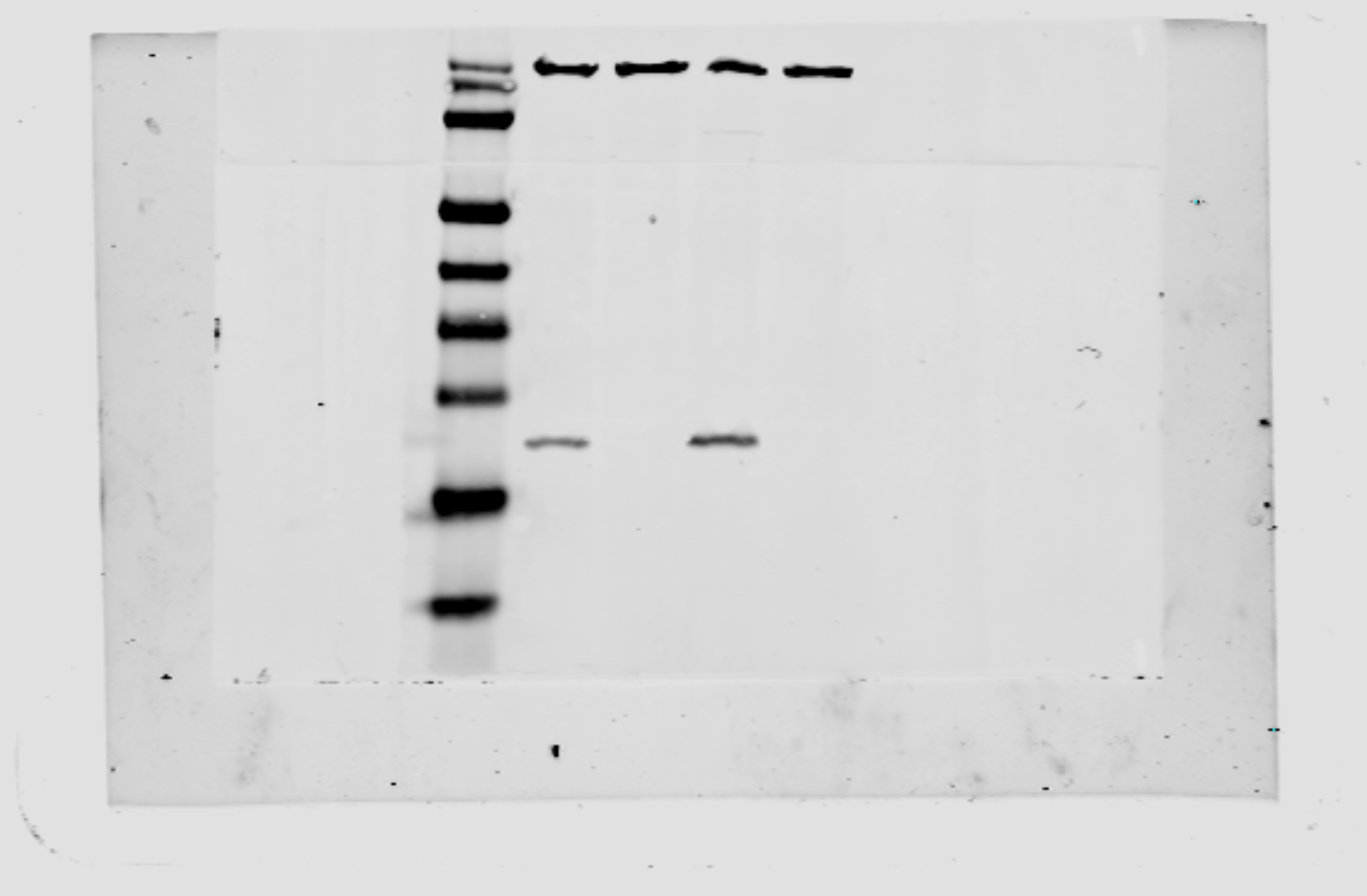

Supplement: Supplementary file 1 [file DataSheet1.zip › Rawdata-RS09070_v2.0/Rawdata-image/Figure 7a.tif]

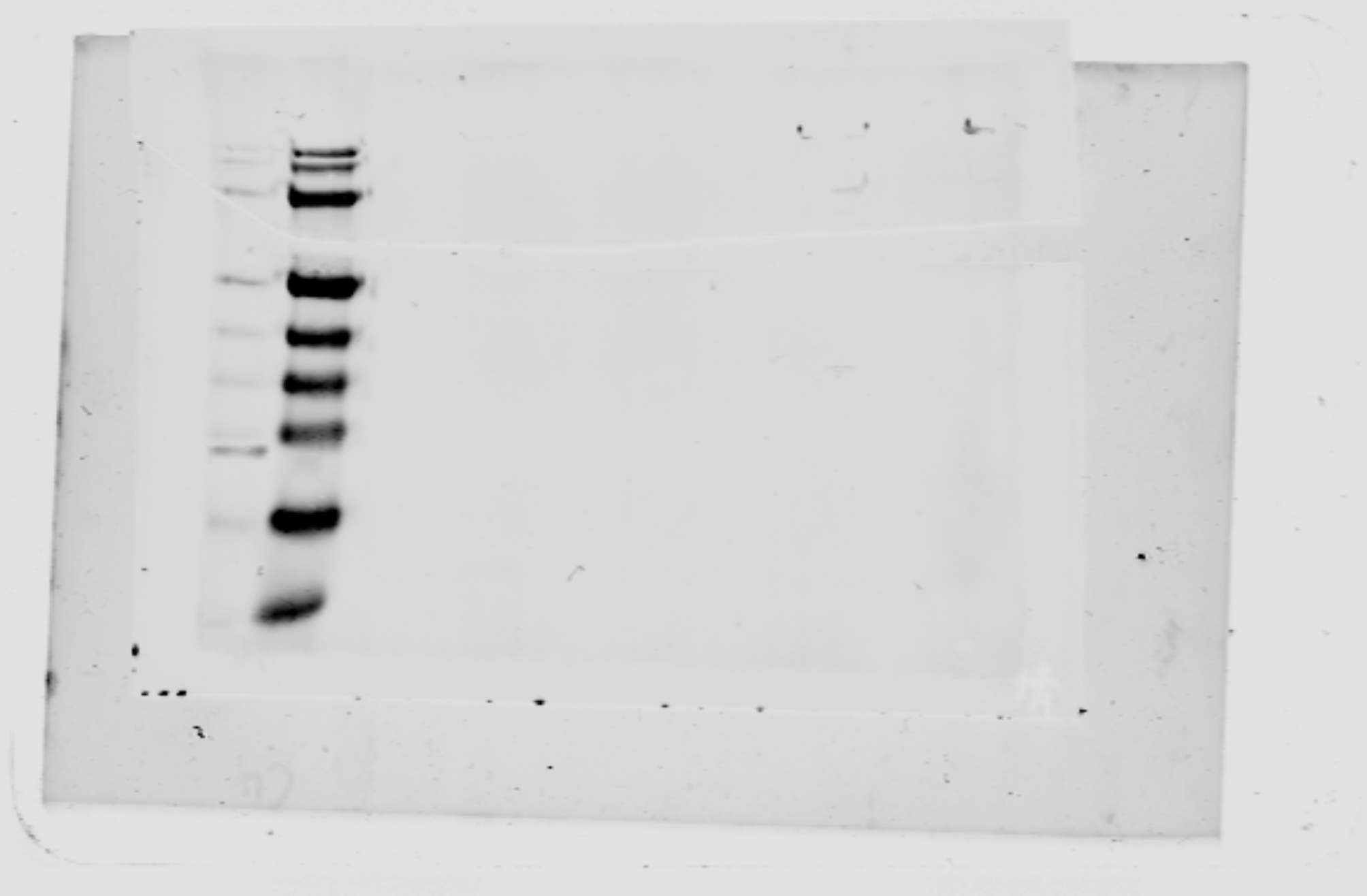

Supplement: Supplementary file 1 [file DataSheet1.zip › Rawdata-RS09070_v2.0/Rawdata-image/Figure 7b.tif]

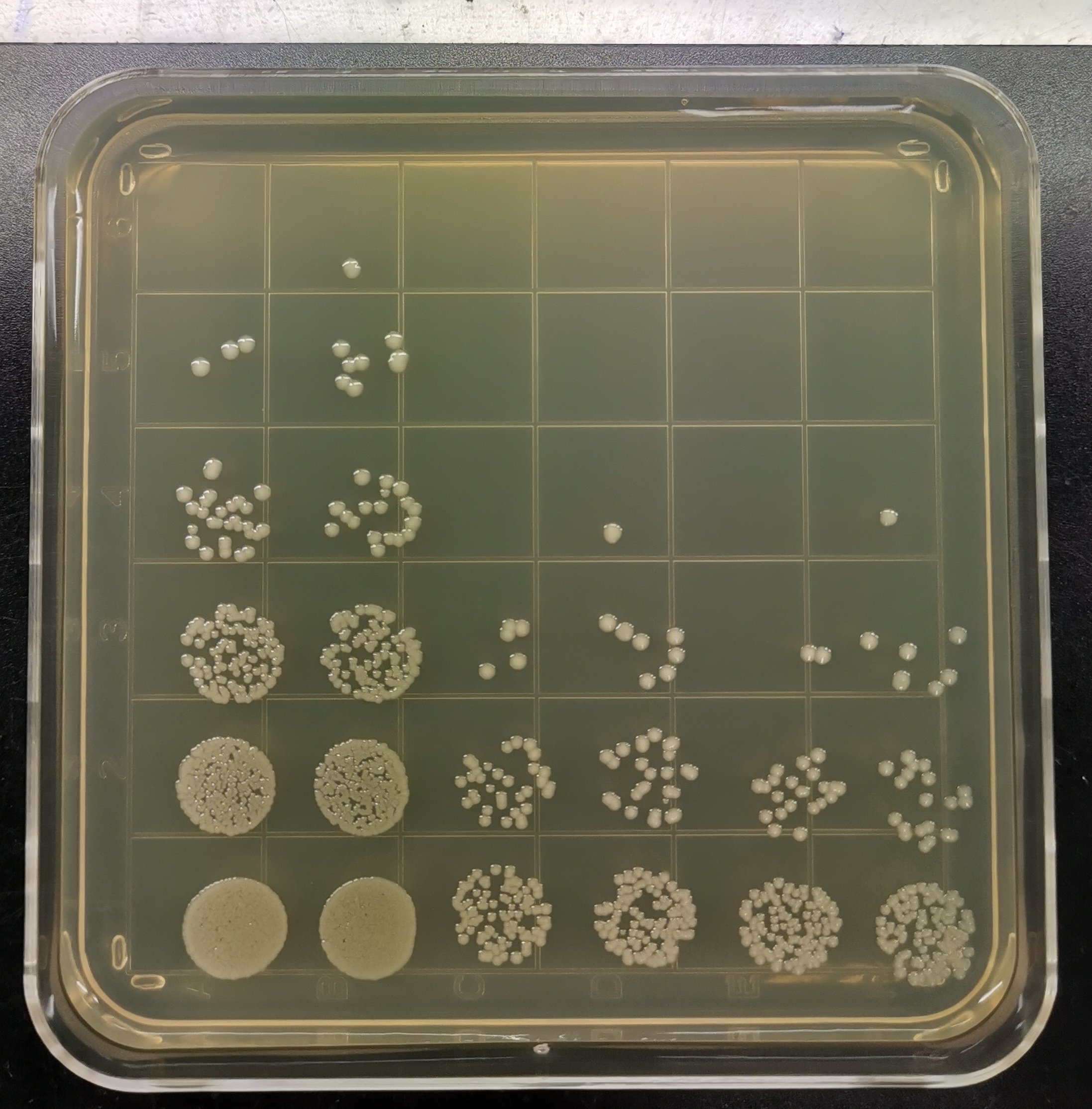

Supplement: Supplementary file 1 [file DataSheet1.zip › Rawdata-RS09070_v2.0/Rawdata-image/Figure 8a.jpg]

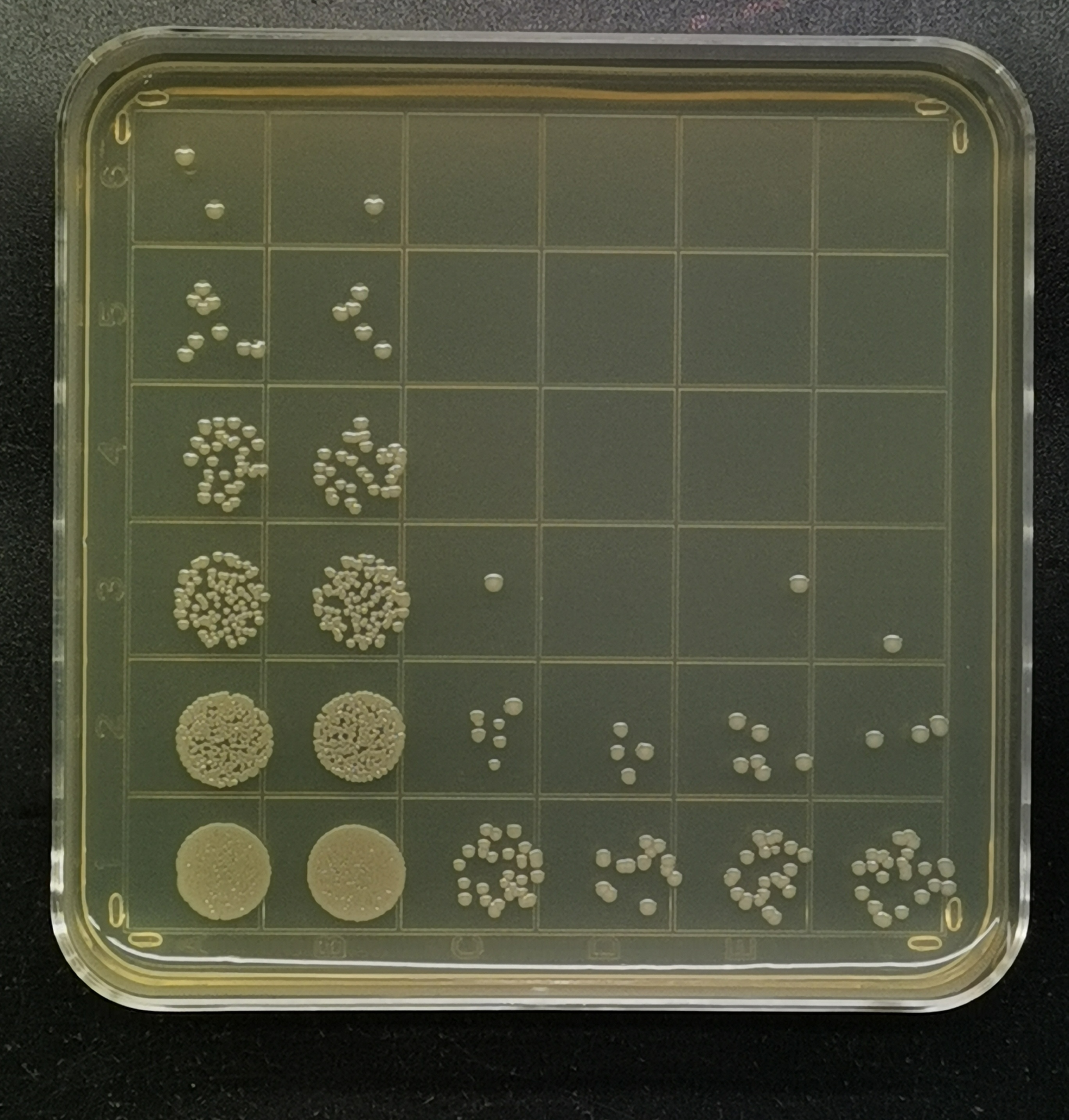

Supplement: Supplementary file 1 [file DataSheet1.zip › Rawdata-RS09070_v2.0/Rawdata-image/Figure 8b.jpg]

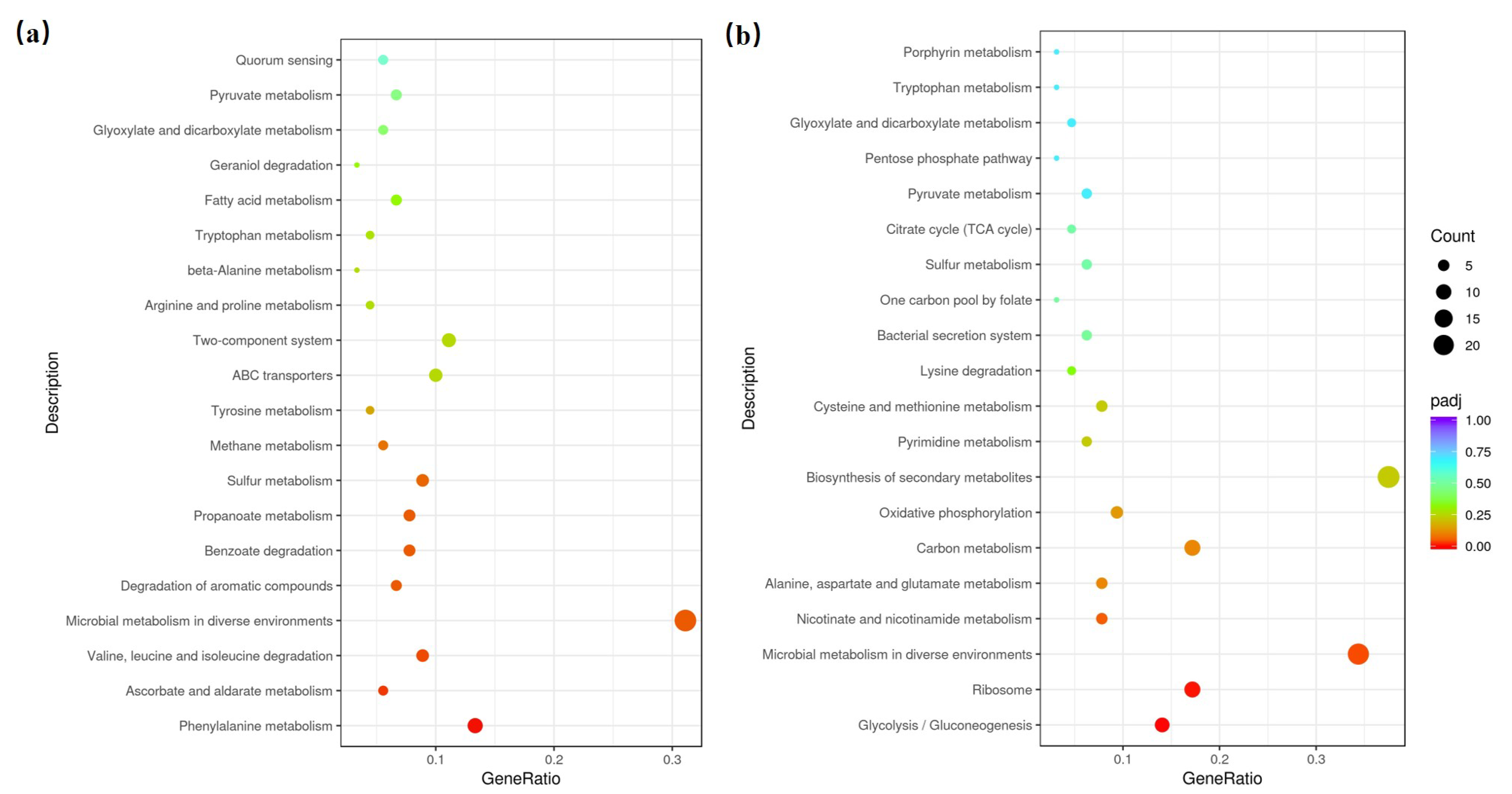

Supplement: Supplementary Figure 1 — KEGG pathway enrichment analysis of TagP-dependent regulated genes based on RNA-seq. KEGG pathway enrichment analysis was performed with a significance threshold of padj < 0.05. The top 20 significantly enriched KEGG pathways of upregulated genes (A) or downregulated genes (B) were selected and depicted in a scatter plot. The x-axis represents the ratio of differentially expressed genes annotated to the KEGG pathway to the total number of differentially expressed genes. The y-axis represents the KEGG pathway, with color shading ranging from purple to red. The intensifying red color indicates a more significant enrichment. Additionally, larger dots represent a higher number of genes enriched in the pathway. [file Image1.tif]

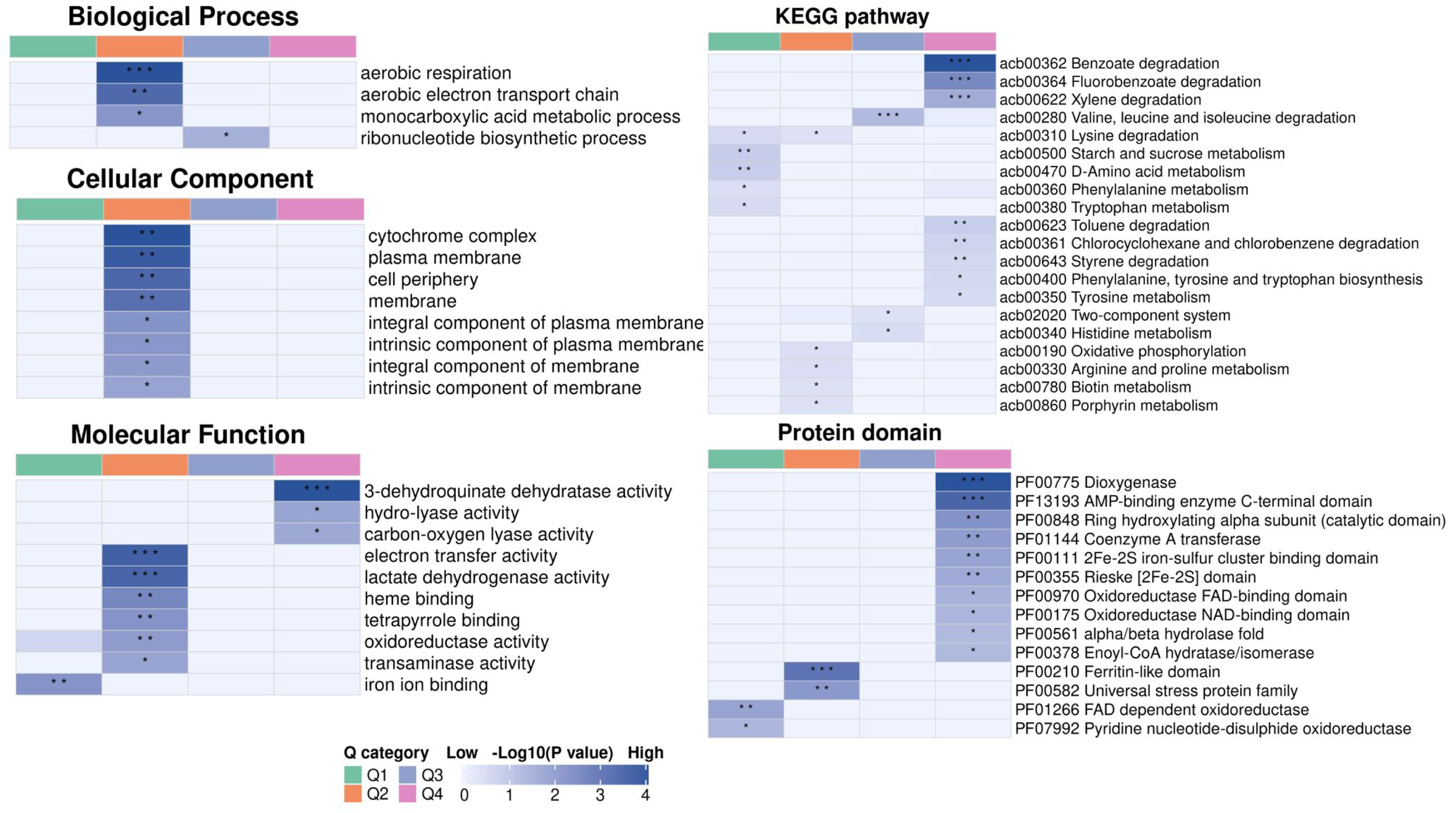

Supplement: Supplementary Figure 2 — Cluster analysis of TagP-dependent regulated proteins based on quantitative proteome. Split the proteins into four groups (Q1 to Q4) based on fold change. Perform GO, KEGG, Reactome, Protein domain, and Wiki Pathways enrichment and functional clustering for each group. Apply hierarchical clustering to group together related functions in different Q groups, using Fisher’s exact test P value as criteria. Generate a heatmap with Q groups displayed horizontally and enriched functions displayed vertically. The intensity of the color blocks represents the significance of enrichment, with blue indicating high significance and blue-white indicating low significance. * denote P < 0.05, ** for P < 0.01, and *** for P < 0.001. [file Image2.tif]

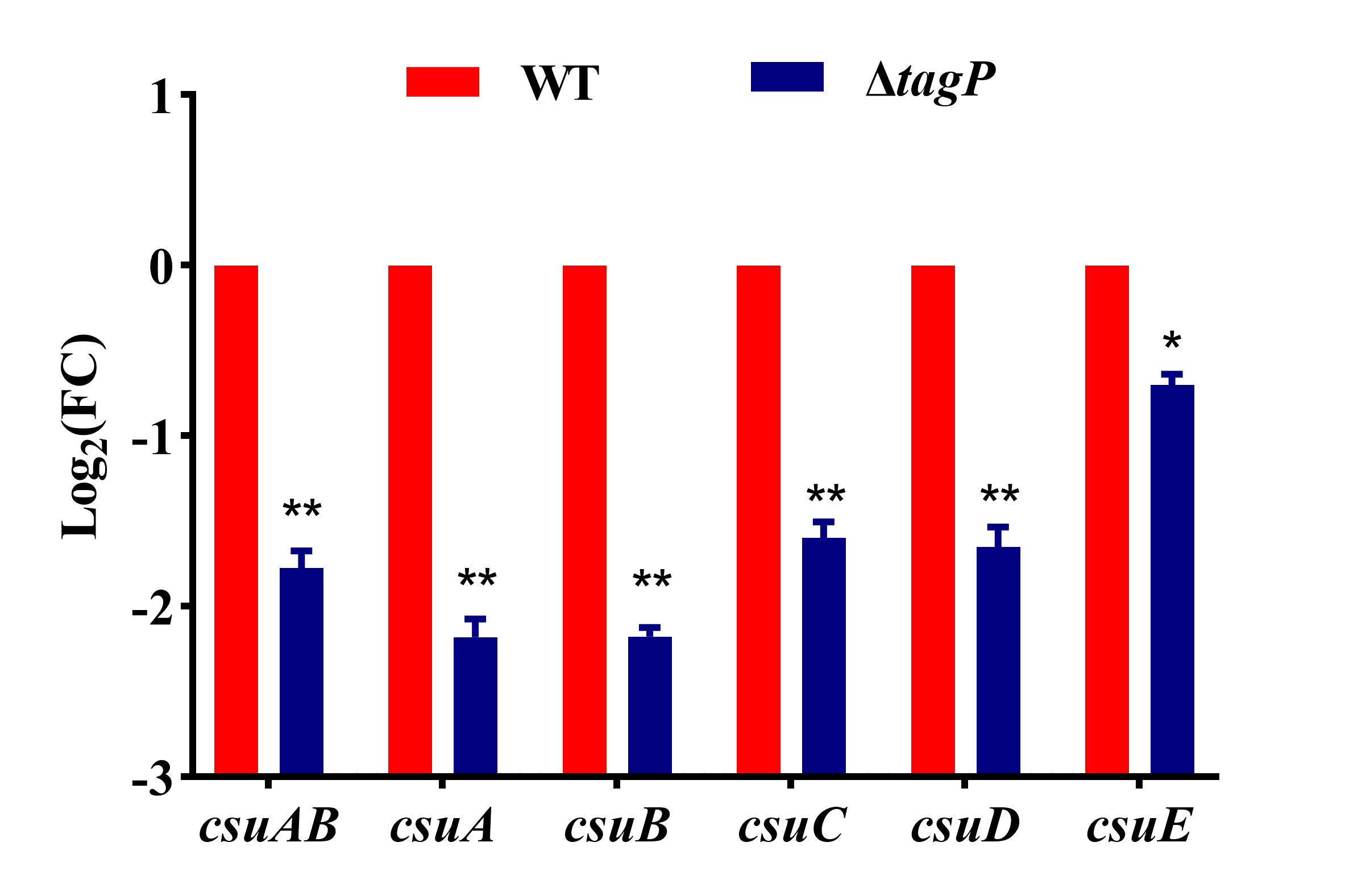

Supplement: Supplementary Figure 3 — qRT–PCR assays for detecting the mRNA in the ATCC 17978 wild-type strains and the tagP null mutant strains. The results shown were the means ± S.D. (n = 3) relative to the wild-type strains results. rpoB was used as the internal control. *, P < 0.05 and **, P < 0.01 compared with wild-type strains based on Student’s t-test. [file Image3.tif]
