# Supplementary material for: TagP, a PAAR-domain containing protein, plays roles in the fitness and virulence of Acinetobacter baumannii
Source: Front Cell Infect Microbiol. 2024 Jul 18;14:1379106. doi: 10.3389/fcimb.2024.1379106 (PMC11348943; doi:10.3389/fcimb.2024.1379106)
Supplement: Supplementary Table 1 — The primers used in this study. [file Table1.docx]

Supplementary Material

# Supplementary Tables

**Supplementary Table 1.** **primers used in this study**

| Primers | Sequences (5'-3') |
| --- | --- |
| 09070upf | GCAAAAATGATCGTCAGTACAGAGA |
| 09070upr | CCAGCCTACACAAACTATTCCTTCTTATTTTCATGAAGTGGTGGAATGATAGCAAAGAGG |
| 09070dnf | AAAGTTATGAATAAATTATTAACGATTC |
| 09070dnr | ATGTTTCTCATCTTCAGTGG |
| Kanf | TGTGTAGGCTGGAGCTGCTTC |
| kanr | CGTTAATAATTTATTCATAACTTTCATATGAATATCCTCCTTA |
| 09070Rvf | AGGCGTCGACTTGATTCCTACAGCGTAGTTTTCT |
| 09070Rvr | CGCGGATCCAATGTGGAACGCGCGGCGT |
| HcpF | CGCCATATGATGAAAGATATATACGTTGAG |
| HcpR | CGCGGATCCTTACGCTGCGTAAGAAGCTGT |

**Supplementary Table 2. The minimal inhibitor concentrations (MICs) of the wild-type and mutant strain to common antibiotics.**

| Antibiotic | MIC (WT) | MIC (Δ*tagP* ) |
| --- | --- | --- |
| CAZ | 2 | 2 |
| FEP | 2 | 2 |
| IMP | ≤0.25 | ≤0.25 |
| MEM | ≤0.25 | ≤0.25 |
| TOB | ≤1 | ≤1 |
| GEN | 8 | 8 |
| AMK | ≤2 | ≤2 |
| CIP | ≤0.25 | ≤0.25 |
| LVX | ≤0.12 | ≤0.12 |
| DOX | ≤0.5 | ≤0.5 |
| MH | ≤1 | ≤1 |
| TGC | ≤0.5 | ≤0.5 |
| COL | ≤0.5 | ≤0.5 |
| TZP | ≤4/1 | ≤4/1 |
| TIM | ≤8/2 | ≤8/2 |
| SXT | 128/32 | 128/32 |

Note: CAZ: Ceftazidime, FEP: cefepime, MEM: Meropenem, IMP: Imipenem, TZP: Piperacillin/tazobactam, TIM: Ticarcillin/Clavulanic acid, PIP: Piperacillin, LVX: Levofloxacin, CIP: Ciprofloxacin, GEN: Gentamicin, TOB: Tobramycin, AMK: Amikacin, SXT: Sulphamethoxazole, Dox: Doxycycline, MH: Minocycline , TGC: Tigecycline, COL: Colistin

**Supplementary Table 4. Primers used for detecting the mRNA of Csu pili genes by qRT-PCR.**

| Primers | Sequences (5'-3') |
| --- | --- |
| q*csuAB*-F | GCAGCTGTTACTGGTCAGGT |
| q*csuAB*-R | CTGTACGTTCACCACCGTCA |
| q*csuA*-F | AATGCGGGTGAAATTGGAGC |
| q*csuA*-R | ACCAGCACACTCGATCTGAA |
| q*csuB*-F | ATGCAGCAGATCCTCAGCTC |
| q*csuB*-R | GGCAAACTTTCCGTACAACGA |
| q*csuC*-F | ATGCGATGGTGCAAATTCGG |
| q*csuC*-R | GCATCTTGCTCGTTGCCATC |
| q*csuD*-F | AAGCCGTTGTGGTTACGACT |
| q*csuD*-R | GTTCGGTGCGTCCTTCTACA |
| q*csuE*-F | TGCCTTCTTTGAGAGCCCTG |
| q*csuE*-R | GGCTCGTTGGTTGCCATTTT |
| q*rpoB*-F | TGTTATTGCACAGGCCGACT |
| q*rpoB*-R | TGCCTGACGTTGCATGTTTG |

**Supplementary Table 5. RNA-seq of genes of the accessory cluster present in *A. b* 17978 UN strain.**

| gene_name | log_2_(FC) | *p*adj | gene_description |
| --- | --- | --- | --- |
| KZA74_09200 | - | - | phage/plasmid replication protein%2C II/X family |
| KZA74_09205 | - | - | helix-turn-helix domain-containing protein |
| KZA74_09210 | - | - | hypothetical protein |
| KZA74_09215 | 0.42 | 0.4691 | hypothetical protein |
| KZA74_09220 | 0.6 | 0.2477 | zonular occludens toxin domain-containing protein |
| KZA74_09225 | 2.82 | 0.0623 | DUF2523 family protein |
| KZA74_09230 | 0.35 | 0.377 | hypothetical protein |
| KZA74_09235 | -0.83 | 0.0132 | hypothetical protein |
| KZA74_09240 | 1.6 | 0.4222 | hypothetical protein |
| KZA74_09245 | - | - | phage/plasmid replication protein%2C II/X family |
| KZA74_09250 | - | - | helix-turn-helix domain-containing protein |
| KZA74_09255 | - | - | hypothetical protein |
| KZA74_09260 | -1.22 | 3.02E-05 | hypothetical protein |
| KZA74_09265 | 0.33 | 0.0002 | fimbrial protein |
| KZA74_09270 | 0.46 | 0.0002 | fimbria/pilus periplasmic chaperone |
| KZA74_09275 | 1.03 | 7.09E-22 | fimbria/pilus outer membrane usher protein |
| KZA74_09280 | 1.01 | 2.18E-08 | fimbrial protein |
| KZA74_09285 | 2.31 | 8.43E-16 | DUF4882 family protein |
| KZA74_09290 | -0.29 | 0.0028 | S-(hydroxymethyl)glutathione dehydrogenase/class III alcohol dehydrogenase |
| KZA74_09295 | -2.17 | 2.8E-109 | hypothetical protein |
| KZA74_09300 | -1.21 | 2.45E-11 | catalase family protein |
| KZA74_09305 | -1.22 | 8.6E-06 | hypothetical protein |
| KZA74_09310 | 0.14 | 0.377 | hypothetical protein |
| KZA74_09315 | 0.94 | 6.4E-19 | phospholipase D family protein |
| KZA74_09320 | 0.48 | 0.0047 | uracil-DNA glycosylase family protein |
| KZA74_09325 | -0.29 | 0.3164 | hypothetical protein |
| KZA74_09330 | 0.02 | 0.9425 | dipeptidase |
| *pqqE* | -0.4 | 0.002 | pyrroloquinoline quinone biosynthesis protein PqqE |
| *pqqD* | -0.19 | 0.3779 | pyrroloquinoline quinone biosynthesis peptide chaperone PqqD |
| *pqqC* | -0.39 | 0.0015 | pyrroloquinoline-quinone synthase PqqC |
| *pqqB* | -0.07 | 0.531 | pyrroloquinoline quinone biosynthesis protein PqqB |
| *pqqA* | 0.25 | 0.6202 | pyrroloquinoline quinone precursor peptide PqqA |
| *ppk1* | 0.86 | 2.43E-15 | polyphosphate kinase 1 |
| KZA74_09365 | -0.5 | 0.0033 | bifunctional metallophosphatase/5'-nucleotidase |
| KZA74_09370 | -0.32 | 0.4683 | transcriptional repressor |
| KZA74_09375 | -0.37 | 0.4429 | isochorismatase family protein |
| KZA74_09380 | 0.09 | 0.8235 | SDR family oxidoreductase |
| KZA74_09385 | 1.4 | 0.0005 | ATP-binding cassette domain-containing protein |
| *modB* | 1.09 | 0.0002 | molybdate ABC transporter permease subunit |
| *modA* | 1.08 | 5.92E-10 | molybdate ABC transporter substrate-binding protein |
| KZA74_09400 | 0.83 | 0.0638 | LysR family transcriptional regulator |
| *antA* | 0.82 | 0.0416 | anthranilate 1%2C2-dioxygenase large subunit |
| *antB* | 0.23 | 0.8185 | anthranilate 1%2C2-dioxygenase small subunit |
| *antC* | 0.29 | 0.4382 | anthranilate 1%2C2-dioxygenase electron transfer component AntC |

“-”: no reads were detected.

## Supplementary Figures


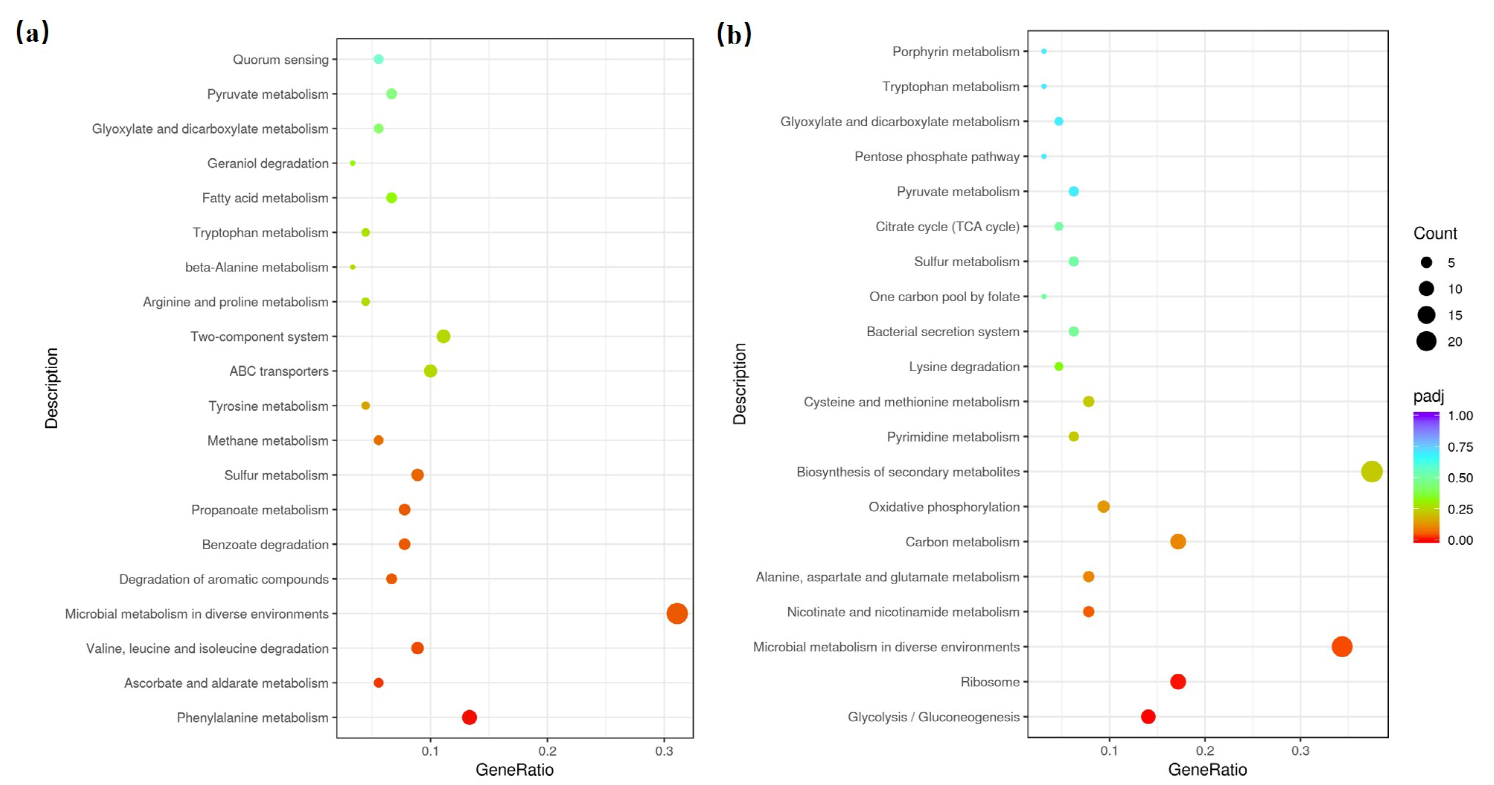


**Supplementary Figure 1.** **KEGG pathway enrichment analysis of TagP-dependent regulated genes based on RNA-seq.** KEGG pathway enrichment analysis was performed with a significance threshold of padj < 0.05. The top 20 significantly enriched KEGG pathways of upregulated genes (a) or downregulated genes (b) were selected and depicted in a scatter plot. The x-axis represents the ratio of differentially expressed genes annotated to the KEGG pathway to the total number of differentially expressed genes. The y-axis represents the KEGG pathway, with color shading ranging from purple to red. The intensifying red color indicates a more significant enrichment. Additionally, larger dots represent a higher number of genes enriched in the pathway.


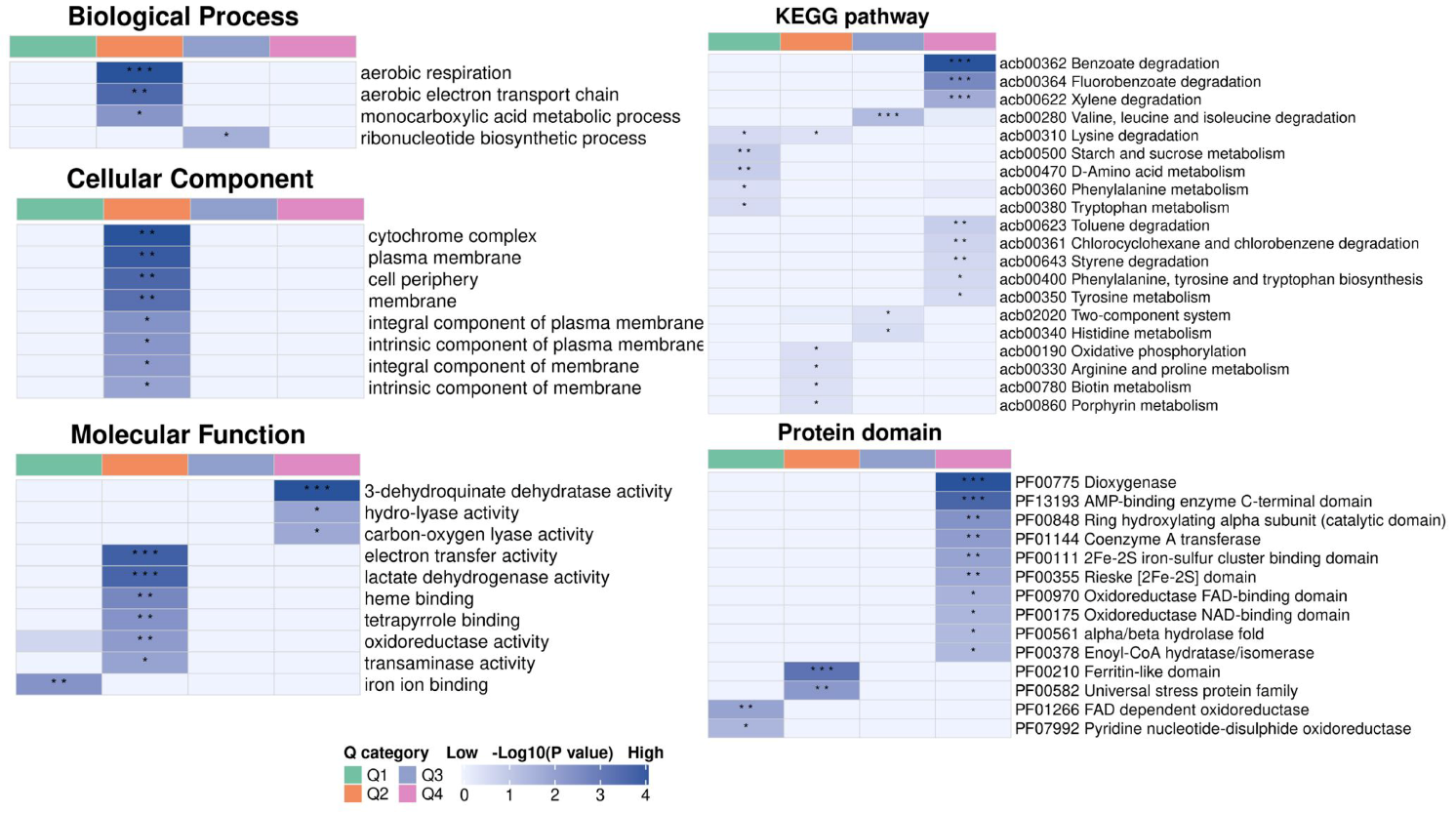


Supplementary Figure 2. Cluster analysis of TagP-dependent regulated proteins based on quantitative proteome. Split the proteins into four groups (Q1 to Q4) based on fold change. Perform GO, KEGG, Reactome, Protein domain, and Wiki Pathways enrichment and functional clustering for each group. Apply hierarchical clustering to group together related functions in different Q groups, using Fisher's exact test *P* value as criteria. Generate a heatmap with Q groups displayed horizontally and enriched functions displayed vertically. The intensity of the color blocks represents the significance of enrichment, with blue indicating high significance and blue-white indicating low significance. * denote *P* < 0.05, ** for *P* < 0.01, and *** for *P* < 0.001.


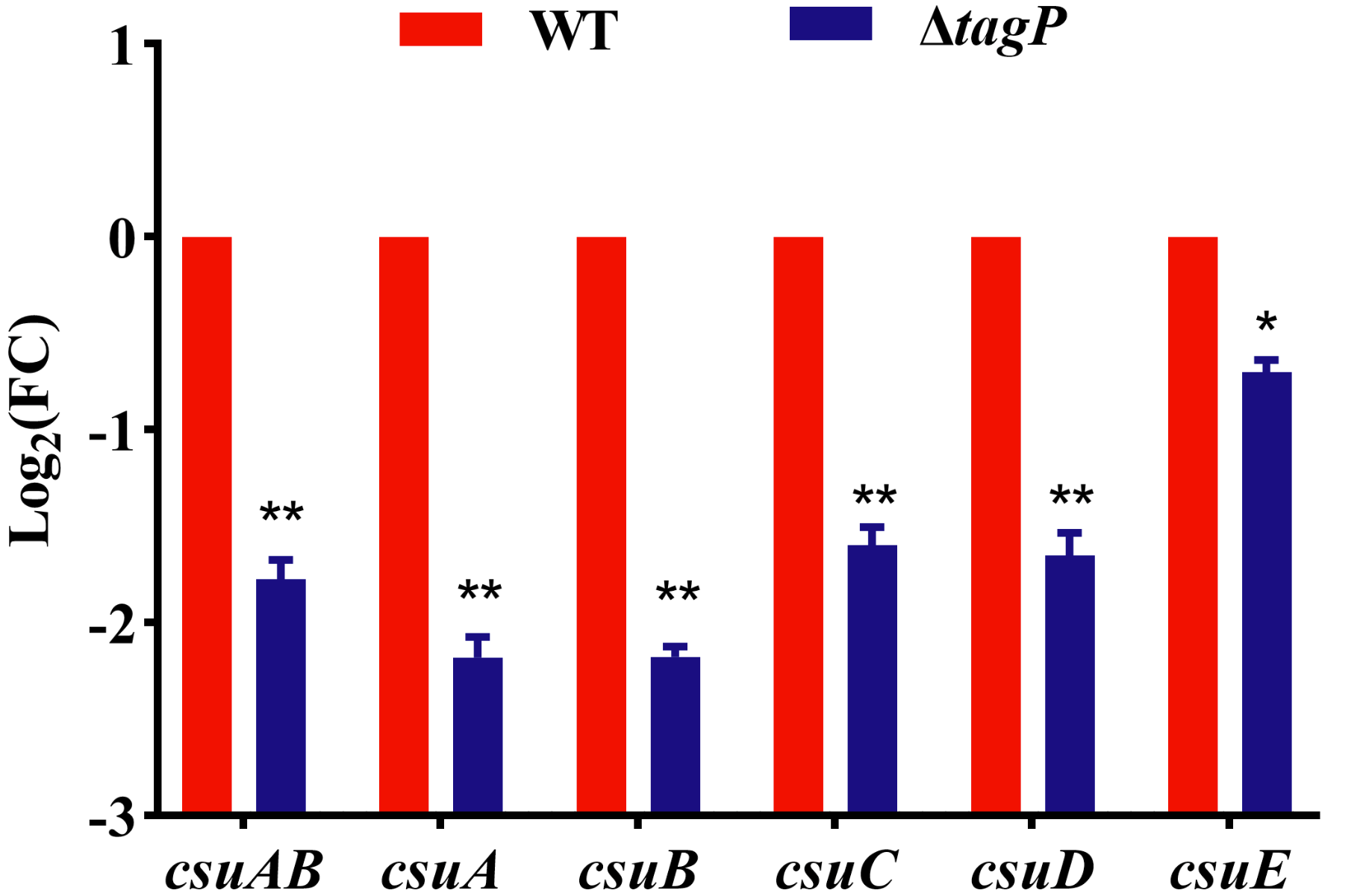


Supplementary Figure 3. qRT–PCR assays for detecting the mRNA in the WT and the *tagP* null mutant strains. The results shown were the means ± S.D. (n = 3) relative to the WT results. *rpoB* was used as the internal control. *, *p* < 0.05 and **, *p* < 0.01 compared with WT based on Student’s t-test.
